# Population structure among octocoral adults and recruits identifies scale dependent patterns of population isolation in The Bahamas

**DOI:** 10.7717/peerj.1019

**Published:** 2015-06-30

**Authors:** Howard R. Lasker, Isabel Porto-Hannes

**Affiliations:** 1 Department of Geology, University at Buffalo, Buffalo, NY, USA; 2 Graduate Program in Evolution, Ecology and Behavior, University at Buffalo, Buffalo, NY, USA

**Keywords:** Microsatellites, Octocorallia, Population genetics, Isolation by distance, Pseudopterogorgia, Gorgonian

## Abstract

Patterns of dispersal and connectivity of the Caribbean gorgonian *Antillogorgia elisabethae* in The Bahamas were assessed in both adults and recently settled recruits from 13 sites using microsatellite loci. Adult populations along the Little Bahama Bank (LBB) exhibited a clear pattern of isolation by distance (IBD) which described 86% of the variance in pairwise genetic distances. Estimates of dispersal based on the IBD model suggested dispersal distances along the LBB on the order of 100 m. Increasing the spatial scale to include sites separated by open ocean generated an apparent IBD signal but the relationship had a greater slope and explained less of the variance. This relationship with distance reflected both stepping stone based IBD and regional differentiation probably created by ocean currents and barriers to dispersal that are correlated with geographic distance. Analysis of recruits from 4 sites on the LBB from up to 6 years did not detect differences between years nor differences with adult populations. The result suggests that neither selection on recruits nor inter-annual variation in dispersal affected adult population structure. Assignment tests of recruits indicated the most likely sources of the recruits were the local or adjacent populations. Most of the patterning in population structure in the northern Bahamas can be explained by geographic distance and oceanographic connectivity. Recognition of these complex patterns is important in developing management plans for *A. elisabethae* and in understanding the effects of disturbance to adult populations of *A. elisabethae* and similar species with limited dispersal.

## Introduction

Sessile and sedentary organisms, such as the invertebrates and fishes found on coral reefs generally have life histories in which reproductive individuals produce larvae that may settle on their natal reef or disperse to other reefs. Those patterns of dispersal control connectivity, the numbers of successful migrants moving between populations, and self-recruitment, the proportion of individuals of local origin. In combination, connectivity and self-recruitment are critical in determining the dynamics of populations, the resilience of metapopulations and the evolution of species. On coral reefs, as for all threatened ecosystems accurate assessments of connectivity are critical components to developing management plans that promote the conservation and resilience of populations (Beger et al., 2014; Jones et al., 2009a; Jones et al., 2009b; Magris et al., 2014; Palumbi, 2003; Van Oppen & Gates, 2006). In this study we use the genetic population structure of both established colonies and recent recruits of the Caribbean octocoral *Antillogorgia elisabethae* to assess connectivity and self-recruitment in The Bahamas.

Connectivity among species such as octocorals is the product of a series of sequential steps starting with the production and dispersal of larvae followed by their settlement and survival (Jones et al., 2009a; Marshall & Morgan, 2011; Zimmer, Fingerut & Zimmer, 2009). Dispersal, the first step in the process, is controlled by traits of the larvae as well as the distribution of suitable habitat and the ocean currents linking habitats (Cowen & Sponaugle, 2009; Jones et al., 2009a; Levin, 2006; Thorrold, Zacherl & Levin, 2007; Zimmer, Fingerut & Zimmer, 2009). Selection acting on the larvae in the water column and among the newly settled recruits (Marshall et al., 2010; Miller, Mundy & Mayfield, 2014), further modulates connectivity. These processes are difficult to directly measure, and population genetic structure and associated measures of gene flow have been used as proxies to characterize connectivity in marine species for over 20 years (Ayre & Hughes, 2004; Doherty, Planes & Mather, 1995; Hellberg, 2007; Hellberg, 2009; Planes, Bonhomme & Galzin, 1993; Purcell et al., 2006; Selkoe et al., 2014 among hundreds of studies). However, as Hellberg (2007) aptly put it, discerning patterns of connectivity from the genetic structure of populations is akin to reading “footprints on water”, and one of the themes of this study is that our ability to discern pattern is affected by the spatial and temporal scales of the study.

Studies of connectivity among coral reef taxa have focused on scleractinian corals (Baums et al., 2012; Souter et al., 2010; Starger et al., 2010; Torda et al., 2013a; Torda et al., 2013b; Underwood et al., 2007; Van Oppen et al., 2011) and fishes (Armsworth, 2002; Cowen et al., 2000; Hogan et al., 2012; Jones et al., 2009a; Pelc et al., 2010; Planes, Parroni & Chauvet, 1998; Puebla, Bermingham & Guichard, 2009; Purcell et al., 2006; Shulman & Bermingham, 1995 among many). Some generalities have been found in analyses across taxa or over ranges of traits such as pelagic larval duration (PLD) (Beger et al., 2014; Holstein, Paris & Mumby, 2014; Selkoe & Toonen, 2011), but beyond broad generalizations, patterns of connectivity are mostly species specific. Investigating a larger and taxonomically more diverse array of species is critical to understanding processes on reefs and for developing ecosystem based management plans.

Octocorals, the focus of this report, are a large and diverse group of sessile marine invertebrates, which are especially abundant on coral reefs and in deeper waters. Branching forms that contain a supporting axis, colloquially known as gorgonians, are abundant on Caribbean coral reefs. Octocorals may be less sensitive to ocean acidification (Gabay, Benayahu & Fine, 2013; Gabay et al., 2014), (but see also Bramanti et al., 2013) and bleaching (Lasker, Peters & Coffroth, 1984; Prada, Weil & Yoshioka, 2010). Thus they may increase in abundance, or at the least increase in relative abundance as scleractinians decline due to acidification and thermal stress (Hoegh-Guldberg et al., 2007; Hughes et al., 2003). Increase in the abundance of octocorals relative to scleractinians has been observed at some sites in the Caribbean (Ruzicka et al., 2013). Octocorals have larvae that are generally larger than scleractinians (Kahng, Benayahu & Lasker, 2011). Thus they may have different patterns of connectivity. An understanding of connectivity among octocoral populations may be critical to understanding the dynamics of an increasingly important member of Caribbean reef communities.

The octocoral *Antillogorgia elisabethae* Bayer (1961) is widely distributed throughout the Caribbean (Bayer, 1961; Kinzie, 1970; Lasker & Coffroth, 1983). Originally described as *Pseudopterogorgia elisabethae*, the species has been reassigned to the *Antillogorgia* as all Caribbean *Pseudopterogorgia* spp. have been reassigned to the resurrected genus *Antillogorgia* (Williams & Chen, 2012). Connectivity of *A. elisabethae* populations is of particular interest as colonies contain a family of natural products, pseudopterosins, which have anti-inflammatory properties (Fenical, 1987; Look et al., 1986b), and the species is harvested in The Bahamas with a reported annual market value of $3–$4 million (Bruckner, 2002). Connectivity among *A. elisabethae* populations should be a key component in the management of the resource.

*Antillogorgia elisabethae* has a reproductive strategy, surface brooding, which is intermediate to brooding and broadcasting spawning. Brooders characteristically release fully competent larvae, while broadcast spawning species release gametes and the resultant larvae often require days to develop, and may spend days, weeks and sometimes months (Graham et al., 2013) in the water column. *A. elisabethae* is gonochoric, and females release eggs to the surface of the colony where they are fertilized and develop into planulae larvae (Gutiérrez-Rodríguez & Lasker, 2004c). The embryos/larvae typically remain on the colony for several days and then are washed off the colony surface. The planulae are negatively buoyant (Gutiérrez-Rodríguez & Lasker, 2004c), but actively swimming planulae have lower sinking rates and have been observed to rise in the water column (unpublished observations). Gutiérrez-Rodríguez & Lasker (2004c) observed planulae that were shaken from a colony surface and found that 13% of the planulae made contact with the substratum within 5 min at distances of 1 to 15 m of the natal colony. Thus 87% of the larvae dispersed a greater, but unknown, distance. Strong wave action, which occasionally occurs during spawning events, can strip embryos from colonies early in their development (HRL, personal observation) and well before they are competent to settle, which raises the possibility of greater dispersal distances.

In previous studies we have established that *A. elisabethae* populations across the northern Bahamas can be partitioned into geographic groups and display what appears to be a pattern of isolation by distance (IBD) (Gutiérrez-Rodríguez, Hannes & Lasker, 2005; Gutiérrez-Rodríguez & Lasker, 2004b). Fine scale comparisons of recruits collected from a single site (Smilansky & Lasker, 2014) suggest that in some years a signal of very local recruitment can be discerned. However, important questions have not been addressed for this and similar species. Do patterns observed among adults simply reflect an equilibrium between local recruitment and immigration, or does selection on recruits maintain the adult pattern? Do patterns of migration (connectivity) vary between years? Comparing patterns in both adults and recruits enabled us to examine those questions, and analysis of the data at different spatial scales also illustrates some of the difficulties of using population structure to characterize the processes controlling connectivity (c.f., Meirmans, 2012).

## Methods

### Sample collection

**Figure 1:**
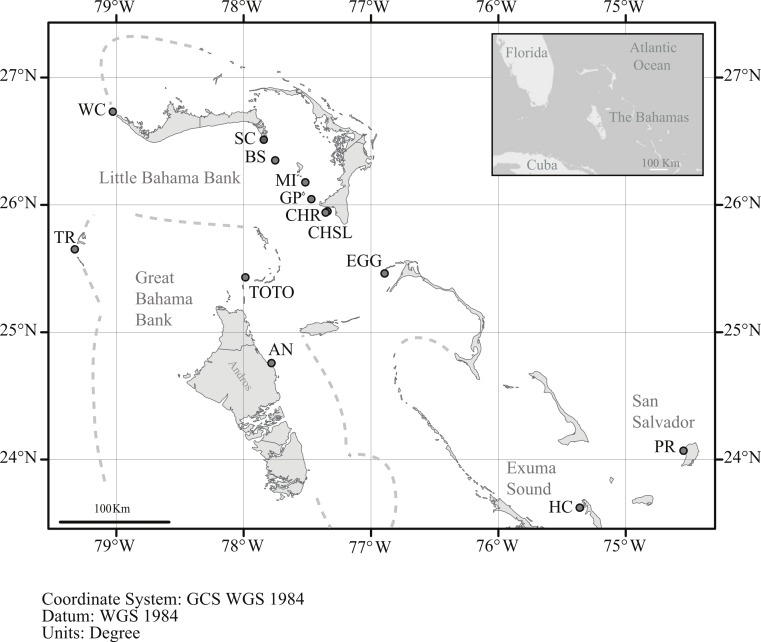
Location of sample sites. Map of the northern Bahamas showing the location of the sample locations. Dashed lines denote the Little and Great Bahama Banks. Map source: World_Light_Gray_Base from Esri, HERE, DeLorme, MapmyIndia, © OpenStreetMap contributors, and the GIS user community. Map created in ArcGIS v. 10.1.

In order to assess genetic diversity and structure of *Antillogorgia elisabethae* tissue samples from adult colonies were collected from 13 sites in the northern Bahamas (Table 1 and Fig. 1). Colony samples were collected from arbitrarily chosen colonies at each site. There have not been any observations of reproduction via fragmentation in detailed surveys for recruits at multiple sites in The Bahamas (Lasker, 2013). Colonies were collected at several meter or greater intervals in order to minimize the likelihood of sampling familial groups. Collections at some sites required searching along paths of 200–400 m. Depth at the different sites ranged from 8 to 22 m. From each colony, ca. 4 cm of live tissue was removed and preserved in 95% ethanol. Many of the sites were similar to those reported in Gutiérrez-Rodríguez & Lasker (2004c), but only two, (San Salvador and Hog Cay) were identical sites and samples.

**Table 1:** *Antillogorgia elisabethae* sampling locations and sample sizes. The number of recruits sampled indicates the total number that were collected and analyzed. Samples were collected in each of 9 years, but sufficient numbers of recruits for population analyses were only collected in some of those years.

Region	Location (Abbreviation)	Latitude	Longitude	Number of adult samples	Number of recruit samples
Little Bahama Bank (LBB)	Wood Cay, Grand Bahama (WC)	26°44.5′	79°02′	58	–
	Sweetings Cay (SC)	26°30.675′	77°50.450′	57	–
	Burrows South (BS)	26°20.957′	77°45.034′	49	70
	Moores Island (MI)	26°10.550′	77°30.875′	55	–
	Gorda Patch Reef (GP)	26°02.641′	77°28.014′	31	61
	Cross Harbour Ridge (CHR)	25°57.341′	77°20.821′	56	508
	Cross Harbour Slope (CHSL)	25°56.936′	77°20.403′	43	137
Great Bahama Bank (GBB)	Triangle Rock, Bimini (TR)	25°39′	79°19.5′	58	–
	Tongue of the Ocean (TOTO)	25°25.784′	77°59.121′	54	–
	Egg Island, Eleuthera (EGG)	25°27.677′	76°53.545′	64	–
	Andros Town, Andros (AN)	24°45.416′	77°46.8′	65	–
San Salvador	Pillar Reef (PR)	24°04.157′	74°32.685′	71	–
Exuma Sound	Hog Cay (HC)	23°37.238′	75°21.591′	43	–

Recruits were collected from a series of 4 sites (Table 1) along the Little Bahama Bank (LBB) every year between 2004 and 2012, with the exception of 2008. At each site all of the recruits within 20 randomly placed 1 m^2^ quadrats were collected and preserved in 95% ethanol. Details of the sites and quadrat placement are presented in Lasker (2013). Collections prior to 2007 were made in May and early November, respectively 5 and 11 months after November/December spawning. In order to collect recruits closer to the time of spawning (and thus subject to less post-settlement mortality, the sampling was changed to January and May/June in 2009 and thereafter. Recruits collected in January were usually single polyps. In both sampling schemes the vast majority of recruits were collected in the first of the two months. All collections were conducted under a research permit from the Department of Marine Resources, The Bahamas.

### Laboratory analysis

Allelic variation was assessed by genotyping a total of 704 samples of *A. elisabethae* adults and 776 recruits. Genomic DNA was isolated from adults or large recruits using 0.5 cm of tissue and from the entire sample among smaller recruits. The CTAB phenol-chloroform-isoamyl alcohol protocol (Coffroth et al., 1992) or DNeasy Tissue Extraction kit (Qiagen) was used for extractions. Loci used in the analyses and references for their use were Pel19, Pel32 (Gutiérrez-Rodríguez & Lasker, 2004a; Gutiérrez-Rodríguez & Lasker, 2004b), Pel62, Pel84 (Lasker et al., 2008) and Ael-AG-1, Ael-AGC-1, Ael-ATC-1, Ael-AGAT-1, Ael-AGAT-2, and Ael-AGAT-3 (Porto-Hannes & Lasker, 2013). In brief, PCR consisted of 10-µL reactions, containing 2 U of *Taq*polymerase, 10 mM Tris–HCl (pH 8.3), 50 mM KCl, 200 µM dNTPs, approximately 10 ng of DNA template, 1.5–2.5 mM MgCl_2_, and 0.025–0.05 µM of each primer. Cycling conditions were 2 min at 94 °C, followed by 28–32 cycles at 94 °C for 30 s, 50–56 °C for 45 s and 72 °C for 45 s, with a final extension period of 10 min at 72 °C.

PCR products were screened on 7% polyacrylamide gels in a LI-COR NEN^®^ Global IR2 DNA Sequencer System, using fluorescently labeled primers as described in Santos et al. (2003). We determined allele sizes by comparing the migration of the PCR products to 50-350 bp size standards (LI-COR Biotechnology Division) regularly spaced across the gel. Scoring was manual with allele sizes frequently verified by comparing migration distances of alleles with those of size standards using ImageJ (NIH).

### Population genetics and statistical analyses

#### Adults

Number of observed (*N_a_*) and effective (*N_e_*) alleles, allele frequencies, information index, observed heterozygosity, unbiased expected heterozygosity and fixation index were determined using GenAlEx v.6.5 (Peakall & Smouse, 2006; Peakall & Smouse, 2012). *F*_IS_ estimates with significance testing based on 1000 randomizations and genetic disequilibrium, based on 15,300 permutations, were determined in FSTAT v.2.9.3 (Goudet, 1995). The presence of null alleles was evaluated with Microchecker v.2.2.3 (Van Oosterhout et al., 2004) and corrected allele frequencies were estimated using FreeNA (Chapuis & Estoup, 2007).

To assess genetic differentiation among populations, estimators of *F*_ST_ were calculated in FSTAT v.2.9.3 (Goudet, 1995) following (Weir & Cockerham, 1984), and *F*_ST_ corrected for potential null alleles was calculated with FreeNA (Chapuis & Estoup, 2007). ${G}_{\mathrm{ST}}^{{\prime\prime}}$, which corrects estimates for bias due to within-population diversity and population sampling bias (Meirmans & Hedrick, 2011) was also calculated in GeneAlex. A Mantel test was performed using GenAlEx to assess whether gene flow patterns followed the isolation by distance model (IBD) (Wright, 1943). Distance was calculated as both linear geographic distance between the sites and adjusted geographic distance which was calculated by measuring the shortest path between sites that neither crossed land nor the shallow water banks, which are generally inhospitable habitats for the species.

Bayesian model-based clustering was used to infer the number of populations (*K*) represented in the samples using Structure v.2.3.4 (Falush, Stephens & Pritchard, 2003; Falush, Stephens & Pritchard, 2007; Pritchard, Stephens & Donnelly, 2000) and the Geneland package (Guillot et al., 2005) in R (R Development Core Team, 2011). As noted in the user manual, Structure is not well suited for populations exhibiting IBD. However, our working hypothesis was that local geography in the Bahamas generates patterns of both IBD and distinct populations. In the Structure analysis, the admixture model and correlated allele frequencies were used (Falush, Stephens & Pritchard, 2003). The length of the burn-in was 100,000 followed by 300,000 MCMC replications. Ten independent replicate runs of *K* values from 1 to 10 were conducted and the results were compiled and displayed using CLUMPP 1.1 (Jakobsson & Rosenberg, 2007) and distruct v.1.1.2 (Rosenberg, 2004). The best estimate of *K* was determined from an examination of the posterior probabilities and with Structure Harvester (Earl & Vonholdt, 2012) following the ad hoc statistic Δ*K* (Evanno, Regnaut & Goudet, 2005).

Geneland uses geographic information to identify the number of populations. The program also incorporates null alleles in the analysis. Missing data were almost always cases in which only a single locus failed to amplify. Therefore, all missing data were treated as null alleles in the Geneland analyses. The Markov chain Monte Carlo (MCMC) repetitions were set at 100,000, thinning to 100, the burn-in period was set at 200 and the correlated allele frequency model was used. Coordinate uncertainty was set to 0.5, decimal degrees. Uncertainty in the coordinates allows samples collected at the same site to be assigned to different populations, otherwise all samples are assigned to the population where they were collected. Different values of the maximum rate of Poisson process (*λ*, from 1 to 100) were used in different test runs in order to find the optimal value. Low *λ* values correspond to weakly fragmented partitions and strong spatial dependence (Guillot et al., 2005). The best K was chosen based on the highest average posterior probability of 10 independent runs. Pattern in the similarity of the populations was also explored using the principal coordinate analysis (PCoA) routine in GenAlEx v.6.5.

#### Recruits

Recruit collections were compared between years, between sites and with adults using both *F*_ST_ and molecular analyses of variance (AMOVA). *F*_ST_ was calculated with both FSTAT and with FreeNA. AMOVA was conducted using ARLEQUIN v.3.5.1.3 (Excoffier, Smouse & Quattro, 1992). These analyses were repeated sequentially, pooling samples across years when no detectable differences were detected and then pooling recruits and adults from the same sites. Assignment tests were conducted using Geneclass2 (Piry et al., 2004), and migration rates were estimated with BayesAss v.1.3 (Wilson & Rannala, 2003). Site and annual patterns in self-recruitment were explored with Chi-square and log-linear analyses. Recruits were also included in additional runs of Structure both without population assignments and with adults assigned to populations corresponding to the collection site.

## Results

### Adults

A total of 704 adult colonies were analyzed from the 13 sites. Summary data for the loci and sites are presented in Table 2 and Table S1. Numbers of alleles ranged from 1 to 43 at individual sites (Table S1) and across all sites ranged from 9 to 50. Heterozygote deficiency was observed at many loci and populations and global tests of Hardy-Weinberg equilibrium (FSTAT v.2.9.3) identified significant deviations from Hardy-Weinberg equilibrium across both sites (not shown) and loci (Table S2). Microchecker identified 2 loci, Pel-62 and Ael-AGAT-1, which had null alleles at 10 of the sites and one locus, Ael-AGAT-2, having null alleles at 9 of the sites. Unless indicated otherwise those 3 loci were not included in subsequent analyses. After dropping the 3 loci with putative null alleles, 23 of 89 site by locus tests for deviation from Hardy-Weinberg equilibrium were significant (*P* ≤ 0.001), but there was no consistent pattern across either loci or sites (Table S2). Examining all combinations of loci and populations, there were 38 cases in which loci were not in genetic equilibrium (*P* ≤ 0.05, Table S3), but none of those cases were significant after probabilities were corrected for multiple comparisons.

**Table 2:** Microsatellite traits for *Antillogorgia elisabethae* adults averaged across 13 populations from The Bahamas.

Microsatellite	*N*	*N_a_*	*N_e_*	*H_o_*	*H_e_*	*F*	*F*_IS_	*F*_IT_	*F*_ST_
Pel-84	50.31(3.39)	9.15(2.88)	2.84(1.31)	0.33(0.06)^**^	0.35(0.07)	0.03(0.03)	0.05	0.275	0.234
Pel-62	49.23(3.30)	3.15(0.60)	1.30(0.06)	0.09(0.02)^**^	0.21(0.03)	0.57(0.11)	0.59	0.61	0.05
Pel-19	51.77(3.38)	5.00(0.51)	2.03(0.19)	0.33(0.04)^**^	0.48(0.03)	0.32(0.07)	0.32	0.45	0.20
Pel-32	50.85(3.19)	4.08(1.47)	1.71(0.47)	0.16(0.07)^**^	0.18(0.08)	0.23(0.10)	0.14	0.36	0.25
Ael-AG	45.31(3.59)	4.08(0.24)	2.16(0.17)	0.37(0.04)^**^	0.50(0.04)	0.26(0.07)	0.27	0.36	0.13
Ael-AGAT1	43.46(3.44)	2.31(0.18)	1.59(0.11)	0.20(0.05)^**^	0.33(0.05)	0.45(0.08)	0.41	0.57	0.27
Ael-AGAT2	38.92(4.65)	1.92(0.18)	1.37(0.09)	0.07(0.02)^**^	0.23(0.05)	0.62(0.09)	0.69	0.82	0.42
Ael-AGAT3	49.31(3.29)	7.08(0.64)	1.73(0.09)	0.39(0.03)^**^	0.40(0.03)	0.03(0.03)	0.03	0.060	0.04
Ael-AGC	49.39(3.31)	6.15(0.55)	3.44(0.38)	0.60(0.06)^**^	0.65(0.06)	0.07(0.03)	0.07	0.19	0.13
Ael-ATC	50.15(3.40)	4.46(0.39)	2.06(0.16)	0.46(0.05)^**^	0.47(0.05)	0.01(0.03)	0.02	0.23	0.24
Combined	47.87(1.12	4.74(0.38)	2.02(0.15)	0.30(0.02)	0.02(0.25)	0.028	0.26	0.40	0.20

**Notes.**

Means (standard errors) of *N* sample size *N_a_* number of observed alleles *N_e_* number of effective alleles *H_o_* observed heterozygosity *H_e_* unbiased expected heterozygosity *F* Fixation index

** significantly lower than expected *p* < 0.001.

Global *F*_ST_ calculated in FSTAT v.2.9.3 was 0.177 and that value as well as the vast majority of pairwise *F*_ST_ values were significantly greater than 0.0 (Table 3). *F*_ST_ calculated with FSTAT v.2.9.3 and FreeNA (Table S4) were very similar to each other. *F*_ST_ values indicate the populations from HC (Exuma Sound) and PR (San Salvador) were clearly differentiated from each other and from all of the other sites. The TOTO and AN sites were also dissimilar to most other sites but to a lesser extent. Although both sites were situated along the western edge of the Tongue of the Ocean, AN was no more similar to TOTO than to the other sites. TR, although separated from the other sites by Andros Is. and the Great Bahama Bank, also had intermediate levels of genetic differentiation to both LBB and GBB sites (*F*_ST_ = 0.0404–0.1469, Table 3). With the exception of the WC population at the western edge of Grand Bahama, comparisons among the populations on the LBB had low (<0.05) pairwise *F*_ST_ values. There were few cases of non-significant *F*_ST_ values and 6 of the 7 such cases were among LBB sites and most commonly between adjacent sites. The seventh case was between EGG and GP, which are not adjacent sites and GP is on the LBB, which is separated from EGG by 60 km at its closest. EGG and all of the LBB sites (excluding WB) had low *F*_ST_ values, with the adjacent CHSL and CHR having particularly low, but significantly greater than 0.0, values of 0.0280 and 0.0296 respectively. As expected from the nature of the index, ${G}_{\mathrm{ST}}^{{\prime\prime}}$ values were greater than *F*_ST_ and are shown in Table S5. One pairwise comparison (GP vs. CHR) in which the *F*_ST_ was not significantly greater than 0.0 had a ${G}_{\mathrm{ST}}^{{\prime\prime}}$ that was significantly greater than 0.

**Table 3:** Pairwise *F*_ST_ among *A. elisabethae* populations in The Bahamas. Site abbreviations as in Table 1. Values in bold are significantly greater than 0.0 at *P* < 0.05. Overall *F*_ST_ = 0.177, 95% confidence interval, 0.117–0.229.

	WC	SC	BS	MI	GP	CHSL	CHR	EGG	TR	TOTO	AN	HC
SC	**0.0786**											
BS	**0.0730**	0.0044										
MI	**0.0653**	**0.0338**	**0.0183**									
GP	**0.0591**	**0.0415**	**0.0255**	0.0049								
CHSL	**0.0655**	**0.0190**	0.0049	**0.0132**	0.0006							
CHR	**0.0834**	**0.0290**	**0.0183**	**0.0233**	0.0072	−0.0001						
EGG	**0.0946**	**0.0809**	**0.0564**	**0.0328**	0.0043	**0.0280**	**0.0296**					
TR	**0.1430**	**0.0648**	**0.0686**	**0.0978**	**0.0815**	**0.0583**	**0.0404**	**0.0846**				
TOTO	**0.1446**	**0.1645**	**0.1515**	**0.1411**	**0.1071**	**0.1044**	**0.1177**	**0.0940**	**0.1469**			
AN	**0.0728**	**0.0783**	**0.0828**	**0.0767**	**0.0567**	**0.0494**	**0.0612**	**0.0745**	**0.1008**	**0.0713**		
HC	**0.2004**	**0.2528**	**0.2565**	**0.2662**	**0.2662**	**0.2510**	**0.2946**	**0.3083**	**0.3445**	**0.2823**	**0.2576**	
PR	**0.3121**	**0.3748**	**0.3746**	**0.4043**	**0.3866**	**0.3664**	**0.4071**	**0.4227**	**0.4385**	**0.3372**	**0.3540**	**0.1853**

**Figure 2:**
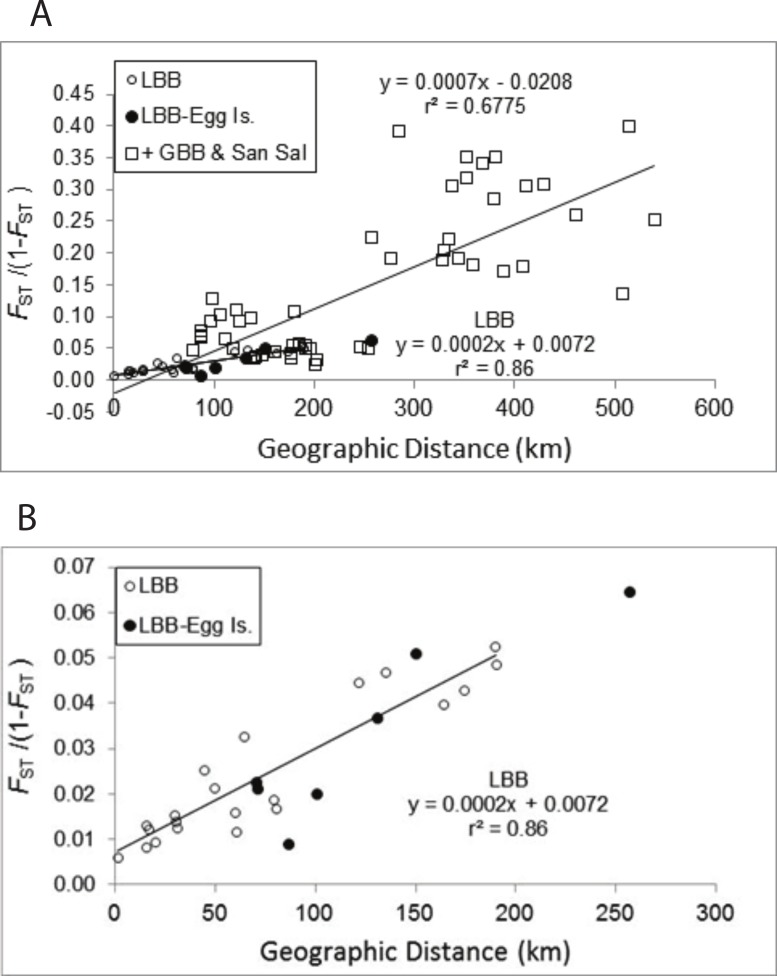
Mantel test of pairwise genetic distance and geographic distance. Relationship between pairwise genetic distance and geographic distance. (A) LBB are comparisons among sites on the Little Bahama Bank; LBB-EGG, comparisons between Little Bahama Bank sites with EGG; + GBB—San Salvador (PR), all other pairwise comparison. The regressions are for the entire data set and for pairwise comparisons among the Little Bahama Bank sites (LBB).

The pattern of increasing genetic differentiation with geographic distance suggested by the *F*_ST_ values is evident in the Mantel tests (Fig. 2A). Plots of genetic distance versus geographic distance were significant (Fig. 2A, *P* = 0.01) and the results were very similar regardless of whether geographic distance or adjusted geographic distance was used (geographic distance, *y* = 0.0007*x* − 0.0208, *R*^2^ = 0.6775, *P* = 0.010; adjusted geographic distance, *y* = 0.0005*x* + 0.0008, *R*^2^ = 0.4857, *P* = 0.010). The proportion of variance explained by the relationship was greater using geographic distance and those data are presented. As evident in Fig. 2B pairwise comparisons among sites from the LBB as well as those comparing LBB sites with EGG form a clear linear pattern. If the regression for the LBB is recalculated including the comparisons with EGG, the slope of the line is unchanged and the regression explains 84% of the variation. The regression that included comparisons among the other sites (Fig. 2A) and of those sites to LBB sites also generated a significant regression, but inspection of the data suggests there are several subsets of comparisons, that do not appear to change with distance. The pairwise comparisons with *F*_ST_/(1 − *F*_ST_) values >0.15 are all comparisons of Exuma Sound (HC) and San Salvador (PR) with the other sites, and each of those sets of comparisons shows little change with distance and perhaps even an inverse relationship.

**Figure 3:**
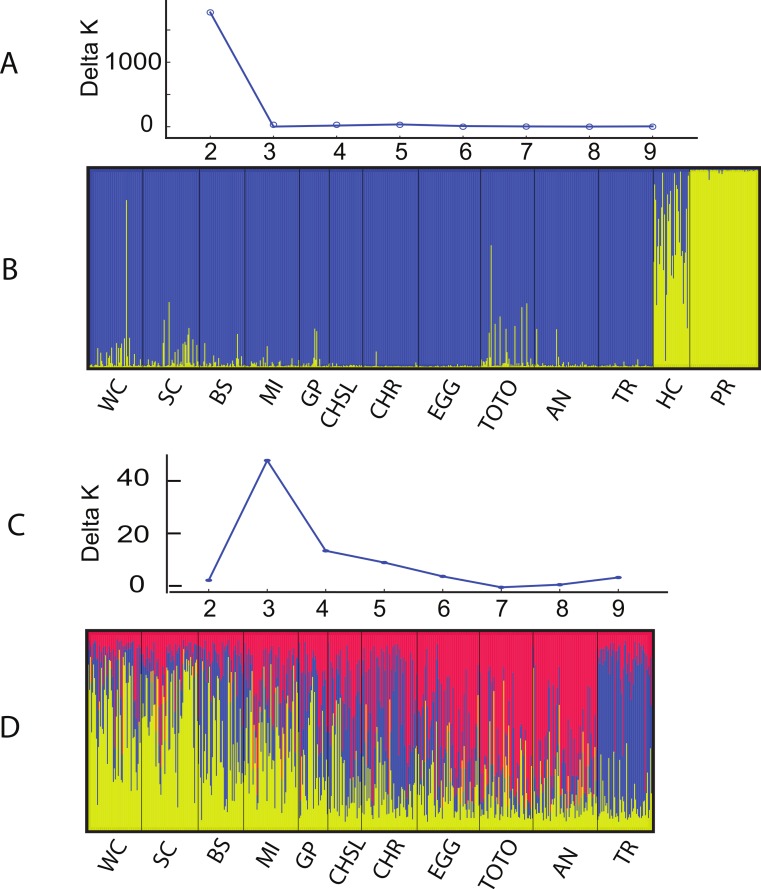
Structure analysis of *A. elisabethae* populations in The Bahamas. Results of Structure analysis. (A) plot of Evanno’s delta K as a function of the number of putative populations for simulations including all 13 sites and 10 loci and (B), inferred population structure. (C) plot of Evanno’s delta K as a function of the number of putative populations for simulations excluding the Exuma Sound and San Salvador populations and D, inferred population structure. Site abbreviations as in Table 1.

The Structure and Geneland analyses of the populations clearly characterized the complex nature of population structure of *A. elisabethae* in The Bahamas. An initial Structure analysis, which included all 13 sites exhibited a best fit model of *K* = 2 (data not shown) in which colonies from Exuma Sound (HC) and San Salvador (PR) generally belonged to one population and those from the other sites to a second population. Subsequent analysis without the Exuma Sound (HC) and San Salvador (PR) collections generated highly variable results. LnP[*k*] values from the Structure analyses did not monotonically increase with increasing *K*, and there were high levels of variance at *K* values >3, both of which suggested the simulations were not converging. Increased steps (200,000 burn in and 10^6^ MCMC replications) had no appreciable effect on the results. Repeating the analysis using all 10 loci; i.e., including those in which null alleles were present, also indicated *K* = 2 when all 13 sites were included (Figs. 3A and 3B). The individuals from San Salvador (PR) belonged to a distinct population with those from Exuma Sound (HC) exhibiting admixture between the San Salvador (PR) population and a second population, which was represented by the colonies from the remaining sites. That analysis suggested *K* = 3 among the 11 sites (Figs. 3C and 3D).

Structure is not well suited for populations that fit an IBD model (Pritchard, Stephens & Donnelly, 2000), and thus the meaning of the specific value of *K* = 3 is unclear. However, the effects of IBD are evident in the manner in which admixture changes between sites in the Structure analysis. The sites in Fig. 3D are arrayed from west to east along the southern edge of LBB and then east to west along the GBB. There was a distinct west to east cline in the contribution of the different “source” populations on the LBB, and individuals from the GBB sites differed from those of the LBB, with the population near Bimini (TR) being the most distinct.

**Figure 4:**
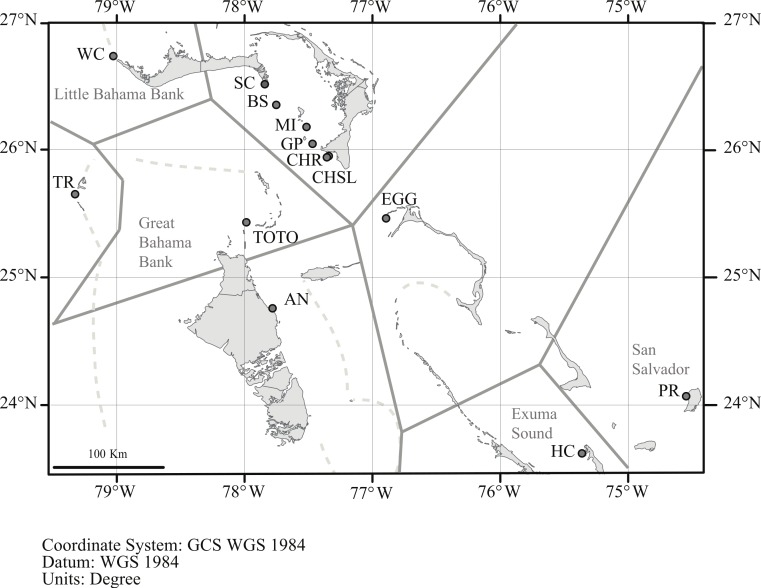
Geneland analysis of *A. elisabethae* populations in The Bahamas. Spatial distribution of estimated populations by Geneland (*K* = 8). Continuous grey lines show the estimated population partitions, and dashed lines depict the edge of the Little Bahama and Great Bahama Banks. Abbreviations as in Table 1. Map source: World_Light_Gray_Base from Esri, HERE, DeLorme, MapmyIndia, © OpenStreetMap contributors, and the GIS user community. Map created in ArcGIS v. 10.1

Geneland incorporates the geographic location of the samples in its analysis, and the analysis partitioned the data into *K* = 8 clusters (Fig. 4). The grouping within the LBB sites and EGG changed with different *λ* values. When *λ* = 2, all LBB sites except WC formed one group. At *λ* = 5–100, WC formed a cluster, BS and SC another cluster and the rest of the LBB populations along with EGG formed a third cluster. At *λ* = 100, the most likely number of cluster was 10, however, two of these clusters were “unsampled” populations. The other 8 clusters were the same as described above. Although the Geneland analysis partitioned the sites into a greater number of populations, the results are concordant with the patterns seen in the Structure plots (Fig. 3). In both analyses there is a core of sites on the LBB. In the Structure analysis, sites on the LBB differed from adjacent sites by relatively small differences in the levels of admixture, while in some Geneland analyses those were further divided into the sites near Great Abaco (CHR, CHSL, GP, MI and sometimes EGG) and those closer to Grand Bahama (SC, BS). WC and TR which had different patterns of admixture in the Structure analysis were also distinct populations in the Geneland analysis.

**Figure 5:**
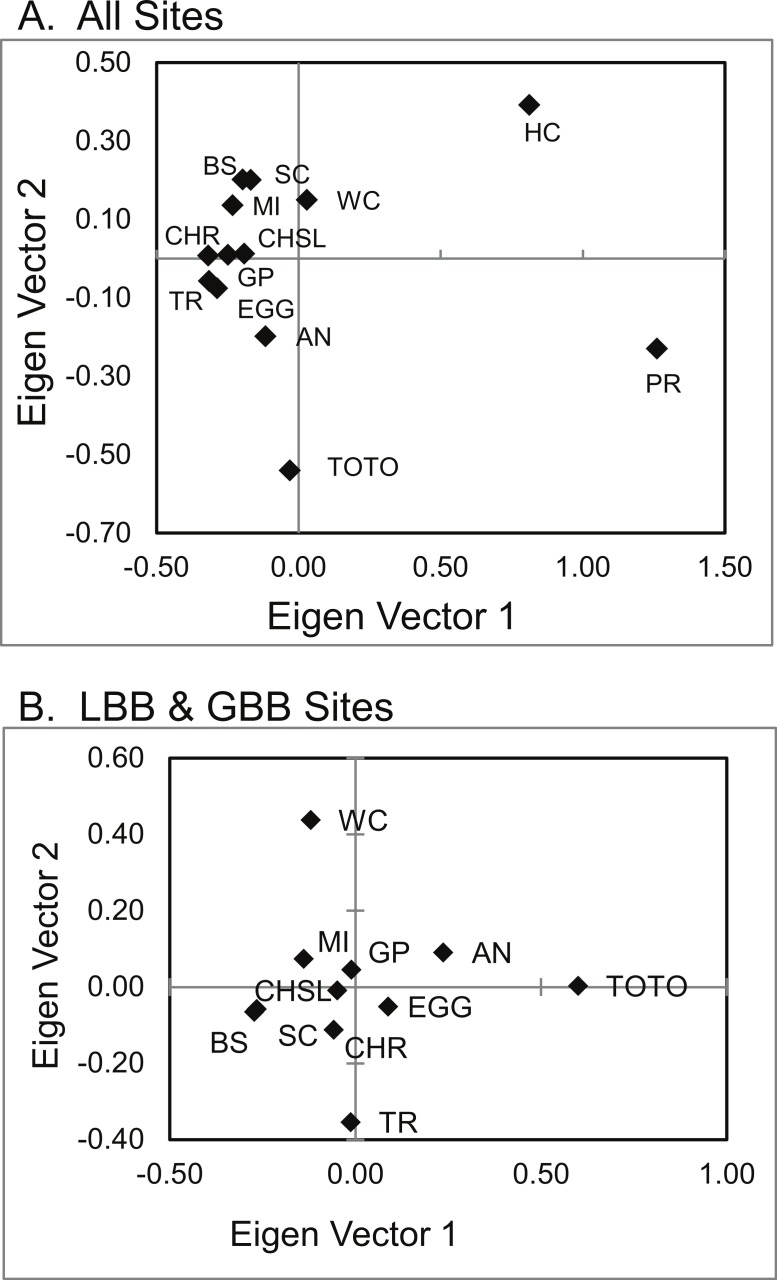
PCoA of genetic distances of *A. elisabethae* in The Bahamas. PCoA of genetic distances for *A. elisabethae* populations from (A) 13 sites in The Bahamas, and (B) an analysis excluding the Exuma Sound (HC) and San Salvador populations (PR).

Principal coordinate analysis (PCoA) of genetic distances (Fig. 5) identified a pattern similar to that hypothesized in Geneland and Structure. San Salvador (PR) and Exuma Sound (HC) and to a lesser extent the Tongue of the Ocean (TOTO) populations were distinct from each other and from the other sites.

#### Recruits

Numbers of recruits collected from the sites were highly variable between years and sites, with collections ranging from 0 to over 100 individuals (Lasker, 2013; Smilansky & Lasker, 2014). In total 776 recruits were used in the analyses. Early in the analyses, when approximately half of the recruits had been genotyped it was recognized that one of the loci, Pel-32, was virtually monomorphic for a 137 bp allele both among the recruit samples and among the LBB adults (recruits, *f*[137] = 0.999; adults *f*[137] = 0.972). As that locus was unlikely to provide useful information in subsequent analyses it was also dropped and the analyses of recruits are based on 6 loci. Statistical comparisons between sites and years were restricted to collections with 20 or more recruits. Pairwise *F*_ST_ (Table 4) between recruits from different years as well as with the adult populations from the sites were very low. The *F*_ST_ values indicate recruit samples were similar to the adults at each collection site and were similar when compared between years. The recruit data closely matched the adults in the relationship between geographic and genetic distance on the LBB. Inclusion of the recruits in the Mantel tests generated a regression with an identical slope as observed among the adult samples and the relationship accounted for 86% of the variance in genetic distance (Fig. S1). Two cases of pairwise *F*_ST_ significantly different from zero were found, between BS recruits from 2004 and 2009 (*F*_ST_ = 0.028) and between the CHSL adults and CHSL 2012 recruits (*F*_ST_ = 0.011). ${G}_{\mathrm{ST}}^{{\prime\prime}}$ also detected an adult—recruit difference at CHSL in 2009 (Table S6). As differences were not reflected in the comparisons of those recruits with those from other years, it would be premature to attribute the differences to recruitment from different sources.

**Table 4:** Pairwise *F*_ST_ of adult and recruit *A. elisabethae* collections from 4 sites in The Bahamas. Sites are identified by location as in Table 1, A or R for adults or recruits, and the year the collection was made. *F*_ST_ values are presented below the diagonal and probability of the value equaling 0.0 is presented above the diagonal. Significant (*p* < 0.05) *F*_ST_ values are denoted in bold. Shaded blocks depict adults and recruits from the same site.

	BS- A- 2005	BS- R- 2004	BS- R- 2009	BS- R- 2012	GP- A- 2004	GP- R- 2004	GP- R- 2005	GP- R- 2009	GP- R- 2012	CHSL- A- 2004	CHSL- R- 2009	CHSL- R- 2012	CHR- A- 2004	CHR- R- 2004	CHR- R- 2007	CHR- R- 2009	CHR- R- 2010	CHR- R- 2011	CHR- R- 2012
BS-A- 2005		.060	.801	.959	.001	.042	.002	.079	.161	.133	.002	.001	.007	.364	.036	.001	.001	.001	.001
BS-R- 2004	.018		.025	.089	.001	.003	.001	.066	.070	.019	.004	.001	.005	.078	.046	.005	.002	.002	.002
BS-R- 2009	.006	**.028**		.802	.006	.023	.002	.042	.241	.182	.001	.001	.003	.118	.014	.001	.001	.003	.002
BS-R- 2012	.005	.023	.008		.019	.134	.004	.262	.389	.494	.007	.004	.078	.609	.158	.029	.049	.017	.018
GP-A- 2004	.023	.045	.026	.024		.924	.183	.760	.924	.182	.051	.022	.032	.011	.113	.045	.128	.163	.023
GP-R- 2004	.023	.045	.030	.024	.009		.301	.873	.660	.606	.190	.370	.404	.121	.409	.308	.313	.472	.473
GP-R- 2005	.037	.054	.044	.041	.022	.025		.285	.246	.066	.183	.074	.070	.017	.190	.103	.060	.192	.131
GP-R- 2009	.018	.028	.023	.018	.009	.010	.022		.756	.720	.436	.307	.451	.242	.675	.604	.614	.646	.852
GP-R- 2012	.025	.039	.025	.025	.012	.020	.032	.016		.596	.280	.199	.184	.202	.512	.405	.491	.610	.416
CHSL-A- 2004	.010	.026	.014	.011	.012	.013	.027	.009	.018		.057	.026	.454	.499	.728	.256	.389	.260	.269
CHSL-R- 2009	.023	.032	.031	.023	.015	.017	.020	.011	.022	.013		.758	.454	.075	.794	.665	.641	.725	.292
CHSL-R- 2012	.023	.035	.033	.023	.013	.012	.020	.011	.022	**.011**	.003		.539	.038	.699	.345	.101	.328	.235
CHR-A- 2004	.013	.027	.022	.014	.013	.012	.019	.009	.021	.006	.006	.004		.142	.973	.932	.816	.774	.772
CHR-R- 2004	.010	.024	.018	.012	.026	.024	.036	.018	.029	.010	.016	.015	.012		.560	.108	.098	.086	.068
CHR-R- 2007	.014	.022	.021	.015	.014	.015	.021	.009	.018	.006	.005	.004	.003	.010		.998	.944	.899	.885
CHR-R- 2009	.014	.023	.022	.015	.011	.013	.017	.007	.017	.006	.003	.003	.002	.011	.001		.779	.877	.839
CHR-R- 2010	.014	.026	.019	.014	.009	.013	.019	.007	.015	.005	.004	.005	.002	.012	.003	.002		.818	.310
CHR-R- 2011	.015	.027	.024	.017	.009	.011	.015	.007	.014	.006	.004	.004	.003	.013	.003	.002	.002		.600
CHR-R- 2012	.014	.024	.021	.015	.012	.011	.017	.005	.016	.006	.005	.004	.003	.012	.003	.002	.003	.002	

Large recruit collections across multiple years were available from CHR (6 y) and CHSL (2y). Those two sites were less than 2 km apart from each other. AMOVA of those data, comparing the two sites with adults and recruits and comparing adults to recruits crossed with years, did not identify significant differences between recruits and adults, between years nor between the two sites (Table S7). Structure analyses in which the recruits are treated as separate sets of samples (results not shown) yield results virtually identical to those using adults alone and most importantly the pattern of admixture observed in the recruits is identical to the adults and varies between sites.

Given the similarity among sites along the LBB it is not surprising that assignments of recruits in Geneclass2 were almost never definitive; 46% of the assignments had probabilities less than 0.5, and only 12% of the assignments had probabilities greater than 0.90. There was no difference in those proportions between the 4 sites (Chi-square test, *P* = 0.93). Log likelihood ratios were used to compare assignments and determine if the best assignment was a statistically (*P* < 0.05) better assignment than the other assignments. Only 2 of the 776 recruits could be definitively be assigned to a single source location. Assignments are presented in Table S8, but as the significance testing indicates second best assignments had only marginally lower probabilities. The mean difference between the probabilities for the best and second best assignments was 0.04. Assignment to the local adult population was not always the most common source of recruits, but the majority of assignments were either to the site the recruit was collected from or one of the nearest sites. Five replicate runs of BayesAss yielded similar results. Those analyses (Table S9) identified significantly greater than 0.0 migration from CHR to MI, GP, CHSL and EGG. All self-recruitment values were significantly greater than 0.0.

The one result from the assignment tests that can be stated with certainty is that there was no evidence of migration between distant sites. While there were a small number of “best assignments” that fell into that category they either had low (<0.5) probabilities and/or assignment probabilities to closer sites were similar. The inability to definitively assign LBB recruits to sites is a consequence of the IBD pattern. Close sites are genetically similar, and thus the probability of accurately assigning individuals to a single site is low.

## Discussion

The major findings of this study are: (1) adult populations along the Little Bahama Bank (LBB) exhibited a clear pattern of isolation by distance (IBD) which described 86% of the variance in pairwise genetic distances, (2) increasing the spatial scale to include sites outside the LBB separated by larger geographic distances and open ocean generated an IBD signal but the relationship is better described as isolated clusters of populations, (3) analysis of recruits from 4 sites on the LBB from up to 6 years did not detect differences between years nor differences with adult populations consistent with the conclusion that the sources of the vast majority of recruits were the local or adjacent populations.

Many marine species have larvae with long life spans and are distributed over broad expanses of ocean. Those traits and the presence of currents that can seemingly carry larvae over long distances suggest that panmixia should be common. Some reef species are undifferentiated across ocean basins (Shulman & Bermingham, 1995), but, as is evident from the many references already cited, finding structured populations is common. For instance, Selkoe et al. (2014) in a survey of 35 marine species along the Hawaiian Archipelago report only 6 cases of panmixia. The remainder of the species exhibited isolation by distance (4), regional clustering (20) and spatially chaotic genetic structure (7). Interpreting findings of panmixia is clear, but distinguishing and explaining the alternative patterns is far more complex. Wright’s stepping stone model; isolation by distance, is an attractive alternative model to panmixia, especially given the often linear nature of reef tracts and island groups. However, the currents connecting reefs within island groups and between islands are not homogeneous. Transit times of larvae, and thus connectivity between stepping stones are likely to be longer (or shorter) than geographic distance suggests. In some cases barriers to dispersal may be present that are not apparent from the geography of reefs alone. Incorporating this more sophisticated concept of “distance” is critical to interpreting the observed patterns of gene flow.

Seascape genetics (Alberto et al., 2011; D’Aloia et al., 2014; Riginos & Liggins, 2013; Selkoe et al., 2010) incorporates the explanatory power of geography and oceanography and incorporates patterns of isolation by barrier (IBB) and isolation by oceanographic distance. In addition, selection can modify these dispersal patterns generating population structure that is strongly affected by isolation by environment (IBE) (Sexton, Hangartner & Hoffmann, 2014). These patterns and processes provide a useful construct for interpreting the population structure of *A. elisabethae* in The Bahamas, where the role of the different processes is dependent on geographic scale.

### Isolation by distance

IBD has been found in a large array of reef species including reef fishes (Buston et al., 2012; Nanninga et al., 2014; Pinsky et al., 2012; Puebla, Bermingham & Guichard, 2009), gastropods (Crandall, Treml & Barber, 2012), octocorals (Andras, Kirk & Harvell, 2011; Ledoux et al., 2010; Yasuda et al., 2014) and scleractinians (Maier et al., 2005; Polato et al., 2010; Porto-Hannes et al., 2015). The pattern of genetic differentiation of *A. elisabethae* along the LBB provides a remarkably good fit to an IBD model, explaining 86% of the variation in genetic differentiation along the LBB. The southern edge of the LBB does not support a continuous well developed reef community. None-the-less, sites that support *A. elisabethae* are apparently common enough to create stepping stone populations, which in concert with *A. elisabethae* larval dispersal generates an IBD model.

*A. elisabethae* on the LBB are distributed in a linear array of populations. In such cases, the slope of the regression (*b* = 2 × 10^−4^, Fig. 2B and Fig. S1) between geographic distance and *F*_ST_/(1 − *F*_ST_) can be used to estimate variance of the dispersal kernel using the equation *b* = 1/(4*Dσ*^2^), where *D* is the population density and *σ*^2^ is the variance of the dispersal kernel (Rousset, 1997). Density of reproductive colonies from 8 sites between Great Abaco and Grand Bahama (CHSL to SC), at which the species was abundant, averages 0.74 m^−2^ (H Lasker, 2011, unpublished data). Assuming the suitable habitat zone is 200 m wide the estimate of density is *D* = 1.48 × 10^5^ km^−1^ of reef tract. If *A. elisabethae* was distributed continuously that would correspond to *σ* of 83 m. However, *A. elisabethae* is patchily distributed along the length of the LBB, with several kilometers of high abundance often followed by kilometers in which the species is virtually absent. In addition, all reproductive size octocoral colonies are not equally successful in either producing or fertilizing eggs (Beiring & Lasker, 2000; Lasker et al., 2008), which further reduces the effective population size. Using an effective density only 1% of the density observed at the study sites only increases the estimate of *σ* to 830 m. The relatively small variance of the dispersal kernel suggests a high level of self-recruitment, which is consistent with the assignment based analyses. This conclusion is also concordant with an analysis of recruitment rates at these and 2 additional LBB sites. In that study the density of reproductive colonies within a 100 m diameter area at each of the 8 sites accounted for 46% of the variation in recruitment between the sites, again suggesting very limited dispersal (Lasker, 2013).

In general, brooding corals exhibit greater population genetic structure and small inferred dispersal distances than broadcast spawning species (Ayre & Dufty, 1994; Hellberg, 1996; Maier et al., 2005; Nishikawa, Katoh & Sakai, 2003; Underwood et al., 2007). *A. elisabethae* is not an internal brooder, but developing embryos/larvae remain on the maternal colony surface for several days (Gutiérrez-Rodríguez & Lasker, 2004c) and are washed off of the colony as fully developed planulae. That should promote larval retention over long distance dispersal. Furthermore, the planulae are negatively buoyant, and 12% of planulae that were shaken from colonies landed on the substratum within a distance of 5 m of the natal colony in one set of observation in 1998 and 14% landed within 15 m of the natal colony during similar observations in 1999 (Gutiérrez-Rodríguez & Lasker, 2004c). Assuming a normal distribution, a value of *σ* of 83 m predicts that 5% of planulae would settle within 5 m and 14% settle within 15 m of the natal colony. Although unambiguous assignments were rare, recruits most commonly were assigned to either the local site or the adjacent site, again suggesting limited dispersal, albeit somewhat greater than that suggested by the IBD analysis.

Finding IBD among *A. elisabethae* populations was also dependent on sampling on an appropriate spatial scale. In this study, the proportion of variance explained by distance declined with the inclusion of more distant sites, which encompassed more complex seascapes. An earlier analysis (Gutiérrez-Rodríguez, Hannes & Lasker, 2005; Gutiérrez-Rodríguez & Lasker, 2004b), with a greater number of sites off of the LBB and using fewer markers identified a significant IBD pattern in the entire data set. However, the overall fit to the data was lower and the pattern among the LBB samples was not recognized. Subsequent reanalysis of those data indicates it was present in those data as well. Rosenberg et al. (2005) suggest that sampling intensity does not have a large effect on discerning structure. However, Selkoe & Toonen (2011) found sampling effects in analyses of IBD in a large survey of the literature on marine species. Similarly a number of authors have noted the importance of scale in differentiating IBD from other spatial patterns and report cases very similar to the *A. elisabethae* data (Meirmans, 2012; Nanninga et al., 2013; Polato et al., 2010; Selkoe et al., 2014; Skoglund et al., 2014; Treml et al., 2012; Tucker et al., 2014; Yasuda et al., 2014).

### Seascape scale processes

Among many marine species simple geographic distance explains only a small portion of the variance in genetic distances, and many authors have adopted approaches emphasizing “seascapes” or “oceanographic distance” (Alberto et al., 2011; Galindo, Olson & Palumbi, 2006; Selkoe et al., 2010; White et al., 2010). Galindo, Olson & Palumbi (2006) found a good fit between the predictions of a biophysical model of larval dispersal with empirical data on the Caribbean coral *Acropora cerviconis.*White et al. (2010) found that consideration of ocean currents and multiple reproductive events could account for over 40% of the genetic differences of a whelk between sites in southern California, and Alberto et al. (2011) found that a measure of current driven connectivity that they called derived oceanographic distance was an excellent predictor of genetic differentiation. Similarly, both scleractinian corals (Baums, Paris & Cherubin, 2006; Foster et al., 2012) and reef fish (Taylor & Hellberg, 2006) exhibit structure predicted from oceanographic models. However, oceanographic distance alone does not offer a complete picture of gene flow. For instance, Foster et al. (2012) found that empirical and modeled estimates of gene flow do not correspond at smaller scales (e.g., Mesoamerican Barrier Reef System), and on a larger geographic and temporal scale, Crandall et al. (2014) concluded that contemporary dispersal predicted from currents was not adequate to explain gene flow of the sea star *Linckia laevigata*.

Among *A. elisabethae* populations in The Bahamas, the presence of IBD regressions with different slopes indicates that once sites away from the LBB were included, connectivity was not a simple function of distance. The degree of isolation between sites, while correlated with distance, probably integrates the effects of geography and current patterns in limiting dispersal. The pattern of IBD observed on the LBB extends across a 70 km open, deep water channel to EGG (Fig. 2B) but breaks down when more distant sites are included (Fig. 2A). Both observations illustrate the importance and potential limitations of seascape scale processes. Connectivity between EGG and the LBB may be driven by currents or eddies carrying larvae across the Northeast Providence Channel (the channel separating the eastern edges of the Little Bahama and Great Bahama Banks, Fig. 1). Alternatively, the population at EGG may have been founded recently and the similarity may reflect that historic event, not ongoing connectivity. In contrast, the other *A. elisabethae* sites appear to be isolated from the LBB, and isolated from each other. Although there was significant correlation between geographic and genetic distance when all of the sites are considered, closer inspection shows the data for the more distant sites (Exuma Sound (HC) and San Salvador (PR) in Fig. 2A) appear as groups of genetically distant sites with no pattern of IBD. Aside from PR (San Salvador) the other sites lie along the perimeter of the GBB, but the large expanse of shallow soft sediment environment of the GBB does not provide suitable habitat for stepping stone populations that could link the sites. Beyond the IBD pattern on the LBB and the distinctness of PR on San Salvador, the structure of the other sites is difficult to decipher. Models of current flow at the time of spawning in The Bahamas may provide further explanatory power in assessing the isolating mechanisms among populations.

Population structure of *A. elisabethae* in The Bahamas, is best described as sets of regional clusters. IBD can be found within areas such as the LBB when the distribution of reefs within those clusters and the sampling intensity is great enough. This illustrates how patterns of differentiation among populations are generated by a mix of IBD along reef tracts and isolating barriers occurring over larger distances and across oceanographic barriers. Nanninga et al. (2014) and Yasuda et al. (2014) reported similar cases in which IBD analysis of geographic subsets of sites generated slopes that differed from each other or from the slope of the combined data set. Polato et al. (2010) also report a similar pattern for the coral *Porites lobata* where a significant IBD pattern was identified as well as a Structure analysis that clearly divided the sites into two populations.

Sampling across multiple scales Ledoux et al. (2010) reported patterns of between region structure and within region IBD that are similar to in *A. elisabethae.* Similarly, the gorgonian *Paramuricea clavata*, which surface broods, exhibited distinct structure between regions across the Mediterranean and, as in our results, an IBD signal within some (Mokhtar-Jamai et al., 2013; Mokhtar-Jamai et al., 2011). The remarkably good fit of the *A. elisabethae* data to the IBD model compared to these other species is likely attributable to the linear pattern of suitable reef habitat along the Little Bahama Bank.

### *Selection and temporal* variation

Genetic analyses of recruits estimates current patterns of gene flow and thus connectivity on an ecological/demographic time scale. The population structure of the *A. elisabethae* recruits closely matched that of the adults, and there was little indication of interanual differences in the pattern. In addition, a finer scale analysis of the same recruits Smilansky & Lasker (2014) found within site spatial autocorrelaton of the recruits in one of 3 years, which suggests that at times very local dispersal may dominate recruitment. These results would be expected in a species with limited dispersal ability such as *A. elisabethae*. Analysis of recruits over 2 to 6 years (CHSL and CHR, respectively) does not preclude the possibilty of unusual events but the similarity of those recruits to adults which live for decades (Goffredo & Lasker, 2005) indicates that the patterns of recruitment that we observed can account for the structure in the adult population. Similarly, the limited variation between years suggests that temporal variability in the delivery of recruits (Fodrie et al., 2011; Hogan et al., 2012) and “sweepstakes” effects (Carson et al., 2010; Pusack et al., 2014) were not occurring among these populations.

Analysis of recruits over multiple years also provides the opportunity to look for evidence of selection modulating the variability in settling recruits. Most of the recruits were collected 1 to 2 months after spawning. Selection against migrant genotypes could have occurred during that time period, but the lack of genetic difference between recruits and adult colonies suggests that maintenance of this pattern was more likely a function of local recruitment than selection limiting connectivity.

### Management implications

The available data suggest that *A. elisabethae* larvae disperse over distances most commonly on the scale of 10^2^ m. Some larvae may settle almost immediately (Gutiérrez-Rodríguez & Lasker, 2004c). The presence of *A. elisabethae* in Bermuda (Look et al., 1986a), demonstrates the distribution of dispersal distances has an extremely long tail, but both genetic (this study) and recruitment rate data suggest the majority of recruits are local in origin. Pinsky et al. (2012) suggest that populations will have closed demographics when the habitat is patchy on a scale >2*σ*, where *σ* is the standard deviation of the dispersal kernel. That describes the environment along the edges of both the Little Bahama and Great Bahama Bank. It suggests that population demographics is highly dependent on self-seeding. *A. elisabethae* is harvested in The Bahamas, with harvesters cropping colonies which are then allowed to recover. The movement of *A. elisabethae* larvae over distances of 10s and 100s of meters but not often further suggests that harvests or naturally occurring disturbances at those or greater scales will depress recruitment and slow recovery of populations. The slow recovery suggested by these patterns may also explain the patchy distribution of *A. elisabethae* where some sites have dense populations while other seemingly similar sites have few or none of the species. These differing densities may be a reflection of the history of the populations and not necessarily the suitability of the sites. Both the frequency and intensity of disturbances to marine systems and particularly coral reefs appears to be increasing (Gardner et al., 2005; Gardner et al., 2003). Understanding patterns of dispersal among marine species cannot reverse those trends but will be crucial to the development of management plans that can enhance recovery between disturbances. In the case of *A. elisabethae* low dispersal distances indicates that self-recruitment is important to the maintenance of populations. Incorporating the correct scaling in management plans will be critical to the maintenance of *A. elisabethae* and similar species.

## Supplemental Information

10.7717/peerj.1019/supp-1 Supplemental Information 1 Supplemental Tables and Figures

10.7717/peerj.1019/supp-2 Supplemental Information 2 Microsatellite data Genotypes of the individuals used in the analyses

## Additional Information and Declarations

### Field Study Permissions

The following information was supplied relating to field study approvals (i.e., approving body and any reference numbers): All collections were conducted under the approval of the Department of Marine Resources, The Bahamas. There is no specific permit number.
