# Supplementary material for: Population structure among octocoral adults and recruits identifies scale dependent patterns of population isolation in The Bahamas
Source: PeerJ. 2015 Jun 30;3:e1019. doi: 10.7717/peerj.1019 (PMC4493681; doi:10.7717/peerj.1019)
Supplement: Supplemental Information 1 [file peerj-03-1019-s001.docx]

Table S1 – Summary Statistics* of microsatellite analyses of adult *A. elisabethae* from populations at 13 sites in The Bahamas using 10 microsatellite loci

|  |  | Locus | | | | | | | | | |
| --- | --- | --- | --- | --- | --- | --- | --- | --- | --- | --- | --- |
| Population | Index | Pel-84 | Pel-62 | Pel-19 | Pel-32 | Ael-AG | Ael-AGAT-1 | Ael-AGAT-2 | Ael-AGAT-3 | Ael-AGC | Ael-ATC |
| Sweetings Cay | N | 57 | 54 | 57 | 50 | 49 | 35 | 46 | 57 | 57 | 57 |
|  | N_a_ | 7 | 2 | 8 | 3 | 5 | 3 | 2 | 9 | 8 | 5 |
|  | N_e_ | 1.550 | 1.077 | 1.751 | 1.106 | 3.116 | 1.368 | 1.830 | 2.045 | 3.187 | 2.087 |
|  | I | 0.845 | 0.158 | 0.979 | 0.224 | 1.241 | 0.483 | 0.646 | 1.131 | 1.404 | 1.060 |
|  | H_o_ | 0.333 | 0.000 | 0.456 | 0.020 | 0.469 | 0.029 | 0.130 | 0.491 | 0.702 | 0.526 |
|  | H_e_ | 0.355 | 0.071 | 0.429 | 0.096 | 0.679 | 0.269 | 0.454 | 0.511 | 0.686 | 0.521 |
|  | uH_e_ | 0.358 | 0.072 | 0.433 | 0.097 | 0.686 | 0.273 | 0.459 | 0.516 | 0.692 | 0.526 |
|  | F | 0.060 | 1.000 | -0.064 | 0.791 | 0.309 | 0.894 | 0.713 | 0.039 | -0.023 | -0.010 |
| Burrows South | N | 46 | 46 | 46 | 46 | 39 | 33 | 31 | 43 | 44 | 44 |
|  | N_a_ | 7 | 2 | 5 | 4 | 5 | 3 | 2 | 7 | 7 | 5 |
|  | N_e_ | 1.416 | 1.375 | 1.446 | 1.337 | 2.518 | 2.252 | 1.661 | 2.015 | 4.177 | 1.908 |
|  | I | 0.715 | 0.445 | 0.680 | 0.519 | 1.102 | 0.910 | 0.587 | 1.001 | 1.621 | 0.964 |
|  | H_o_ | 0.283 | 0.326 | 0.196 | 0.283 | 0.462 | 0.152 | 0.032 | 0.535 | 0.727 | 0.409 |
|  | H_e_ | 0.294 | 0.273 | 0.308 | 0.252 | 0.603 | 0.556 | 0.398 | 0.504 | 0.761 | 0.476 |
|  | uH_e_ | 0.297 | 0.276 | 0.312 | 0.255 | 0.611 | 0.565 | 0.405 | 0.510 | 0.769 | 0.481 |
|  | F | 0.039 | -0.195 | 0.366 | -0.122 | 0.234 | 0.727 | 0.919 | -0.062 | 0.044 | 0.141 |
| Wood Cay | N | 50 | 54 | 54 | 54 | 51 | 46 | 45 | 53 | 52 | 50 |
|  | N_a_ | 9 | 3 | 4 | 1 | 4 | 3 | 2 | 10 | 7 | 7 |
|  | N_e_ | 2.019 | 1.751 | 1.736 | 1.000 | 1.954 | 2.027 | 1.750 | 2.342 | 4.292 | 3.388 |
|  | I | 1.119 | 0.768 | 0.708 | 0.000 | 0.875 | 0.806 | 0.620 | 1.210 | 1.604 | 1.419 |
|  | H_o_ | 0.520 | 0.056 | 0.130 | 0.000 | 0.490 | 0.152 | 0.222 | 0.491 | 0.750 | 0.700 |
|  | H_e_ | 0.505 | 0.429 | 0.424 | 0.000 | 0.488 | 0.507 | 0.429 | 0.573 | 0.767 | 0.705 |
|  | uH_e_ | 0.510 | 0.433 | 0.428 | 0.000 | 0.493 | 0.512 | 0.433 | 0.578 | 0.774 | 0.712 |
|  | F | -0.031 | 0.870 | 0.694 | #N/A | -0.004 | 0.700 | 0.482 | 0.144 | 0.022 | 0.007 |
| Mores Is. | N | 52 | 48 | 55 | 55 | 51 | 39 | 39 | 55 | 55 | 54 |
|  | N_a_ | 5 | 4 | 6 | 2 | 4 | 2 | 2 | 7 | 8 | 7 |
|  | N_e_ | 1.412 | 1.237 | 1.464 | 1.018 | 1.654 | 1.385 | 1.385 | 1.743 | 4.133 | 2.375 |
|  | I | 0.650 | 0.418 | 0.685 | 0.052 | 0.730 | 0.451 | 0.451 | 0.940 | 1.618 | 1.244 |
|  | H_o_ | 0.288 | 0.083 | 0.291 | 0.018 | 0.392 | 0.128 | 0.026 | 0.400 | 0.764 | 0.593 |
|  | H_e_ | 0.292 | 0.192 | 0.317 | 0.018 | 0.395 | 0.278 | 0.278 | 0.426 | 0.758 | 0.579 |
|  | uH_e_ | 0.294 | 0.194 | 0.320 | 0.018 | 0.399 | 0.281 | 0.281 | 0.430 | 0.765 | 0.584 |
|  | F | 0.011 | 0.565 | 0.082 | -0.009 | 0.008 | 0.538 | 0.908 | 0.061 | -0.007 | -0.024 |
| Gorda Patch Reef | N | 30 | 30 | 30 | 30 | 28 | 22 | 14 | 28 | 26 | 29 |
|  | N_a_ | 7 | 3 | 6 | 1 | 3 | 2 | 3 | 7 | 7 | 5 |
|  | N_e_ | 1.325 | 1.356 | 2.171 | 1.000 | 1.446 | 1.816 | 1.806 | 1.467 | 4.727 | 2.507 |
|  | I | 0.624 | 0.502 | 1.121 | 0.000 | 0.589 | 0.642 | 0.787 | 0.763 | 1.706 | 1.176 |
|  | H_o_ | 0.267 | 0.133 | 0.500 | 0.000 | 0.143 | 0.318 | 0.143 | 0.250 | 0.577 | 0.517 |
|  | H_e_ | 0.246 | 0.263 | 0.539 | 0.000 | 0.309 | 0.449 | 0.446 | 0.318 | 0.788 | 0.601 |
|  | uH_e_ | 0.250 | 0.267 | 0.549 | 0.000 | 0.314 | 0.460 | 0.463 | 0.324 | 0.804 | 0.612 |
|  | F | -0.086 | 0.493 | 0.073 | #N/A | 0.537 | 0.292 | 0.680 | 0.214 | 0.268 | 0.139 |
| Cross Harbour Slope | N | 29 | 29 | 33 | 33 | 31 | 29 | 26 | 31 | 33 | 31 |
|  | N_a_ | 3 | 3 | 3 | 2 | 3 | 2 | 2 | 6 | 7 | 4 |
|  | N_e_ | 1.279 | 1.410 | 1.855 | 1.062 | 2.008 | 1.665 | 1.122 | 1.870 | 3.654 | 2.119 |
|  | I | 0.441 | 0.516 | 0.799 | 0.136 | 0.865 | 0.589 | 0.221 | 0.964 | 1.548 | 0.983 |
|  | H_o_ | 0.172 | 0.069 | 0.394 | 0.000 | 0.323 | 0.345 | 0.115 | 0.484 | 0.636 | 0.452 |
|  | H_e_ | 0.218 | 0.291 | 0.461 | 0.059 | 0.502 | 0.400 | 0.109 | 0.465 | 0.726 | 0.528 |
|  | uH_e_ | 0.222 | 0.296 | 0.468 | 0.060 | 0.510 | 0.407 | 0.111 | 0.473 | 0.738 | 0.537 |
|  | F | 0.210 | 0.763 | 0.145 | 1.000 | 0.358 | 0.137 | -0.061 | -0.040 | 0.124 | 0.145 |
| Cross Harbour Ridge | N | 56 | 56 | 56 | 56 | 55 | 55 | 44 | 56 | 56 | 55 |
|  | N_a_ | 8 | 3 | 4 | 1 | 4 | 2 | 1 | 6 | 7 | 5 |
|  | N_e_ | 1.327 | 1.611 | 1.858 | 1.000 | 2.143 | 1.541 | 1.000 | 1.702 | 3.630 | 2.129 |
|  | I | 0.628 | 0.601 | 0.788 | 0.000 | 0.925 | 0.536 | 0.000 | 0.848 | 1.547 | 1.018 |
|  | H_o_ | 0.250 | 0.071 | 0.321 | 0.000 | 0.436 | 0.236 | 0.000 | 0.446 | 0.750 | 0.618 |
|  | H_e_ | 0.246 | 0.379 | 0.462 | 0.000 | 0.533 | 0.351 | 0.000 | 0.412 | 0.724 | 0.530 |
|  | uH_e_ | 0.249 | 0.383 | 0.466 | 0.000 | 0.538 | 0.354 | 0.000 | 0.416 | 0.731 | 0.535 |
|  | F | -0.015 | 0.812 | 0.304 | #N/A | 0.182 | 0.327 | #N/A | -0.083 | -0.035 | -0.166 |
| Bimini | N | 54 | 54 | 55 | 55 | 47 | 52 | 17 | 50 | 49 | 49 |
|  | N_a_ | 5 | 2 | 3 | 2 | 4 | 2 | 1 | 3 | 8 | 3 |
|  | N_e_ | 1.259 | 1.338 | 2.260 | 1.018 | 3.012 | 1.649 | 1.000 | 1.818 | 3.082 | 1.364 |
|  | I | 0.476 | 0.419 | 0.886 | 0.052 | 1.137 | 0.582 | 0.000 | 0.789 | 1.512 | 0.505 |
|  | H_o_ | 0.222 | 0.074 | 0.418 | 0.018 | 0.702 | 0.269 | 0.000 | 0.540 | 0.612 | 0.306 |
|  | H_e_ | 0.205 | 0.252 | 0.558 | 0.018 | 0.668 | 0.393 | 0.000 | 0.450 | 0.676 | 0.267 |
|  | uH_e_ | 0.207 | 0.255 | 0.563 | 0.018 | 0.675 | 0.397 | 0.000 | 0.454 | 0.683 | 0.270 |
|  | F | -0.082 | 0.707 | 0.250 | -0.009 | -0.051 | 0.316 | #N/A | -0.201 | 0.094 | -0.148 |
| NW Tongue of the Ocean | N | 54 | 44 | 54 | 54 | 52 | 46 | 36 | 49 | 54 | 51 |
|  | N_a_ | 8 | 2 | 5 | 4 | 5 | 2 | 2 | 6 | 5 | 4 |
|  | N_e_ | 1.489 | 1.046 | 1.885 | 1.141 | 1.503 | 1.431 | 1.528 | 1.547 | 2.003 | 1.999 |
|  | I | 0.791 | 0.108 | 0.904 | 0.302 | 0.714 | 0.479 | 0.530 | 0.777 | 0.907 | 0.814 |
|  | H_o_ | 0.296 | 0.000 | 0.352 | 0.093 | 0.288 | 0.196 | 0.111 | 0.347 | 0.389 | 0.588 |
|  | H_e_ | 0.329 | 0.044 | 0.469 | 0.124 | 0.335 | 0.301 | 0.346 | 0.354 | 0.501 | 0.500 |
|  | uH_e_ | 0.332 | 0.045 | 0.474 | 0.125 | 0.338 | 0.305 | 0.351 | 0.357 | 0.505 | 0.505 |
|  | F | 0.098 | 1.000 | 0.251 | 0.251 | 0.138 | 0.351 | 0.679 | 0.019 | 0.223 | -0.177 |
| Egg Is. | N | 60 | 57 | 63 | 59 | 59 | 48 | 59 | 59 | 51 | 61 |
|  | N_a_ | 3 | 2 | 4 | 2 | 3 | 2 | 1 | 8 | 7 | 4 |
|  | N_e_ | 1.069 | 1.170 | 1.957 | 1.017 | 1.394 | 1.064 | 1.000 | 1.651 | 6.193 | 2.090 |
|  | I | 0.165 | 0.276 | 0.769 | 0.049 | 0.551 | 0.139 | 0.000 | 0.930 | 1.868 | 0.977 |
|  | H_o_ | 0.067 | 0.018 | 0.238 | 0.017 | 0.220 | 0.021 | 0.000 | 0.356 | 0.863 | 0.475 |
|  | H_e_ | 0.065 | 0.145 | 0.489 | 0.017 | 0.283 | 0.061 | 0.000 | 0.394 | 0.839 | 0.521 |
|  | uH_e_ | 0.065 | 0.147 | 0.493 | 0.017 | 0.285 | 0.061 | 0.000 | 0.398 | 0.847 | 0.526 |
|  | F | -0.028 | 0.879 | 0.513 | -0.009 | 0.221 | 0.656 | #N/A | 0.097 | -0.029 | 0.088 |
| Andros | N | 63 | 62 | 65 | 64 | 63 | 57 | 60 | 65 | 64 | 65 |
|  | N_a_ | 5 | 3 | 4 | 4 | 5 | 3 | 2 | 6 | 3 | 3 |
|  | N_e_ | 1.177 | 1.214 | 2.003 | 1.288 | 1.843 | 1.262 | 1.123 | 1.688 | 1.966 | 2.202 |
|  | I | 0.379 | 0.346 | 0.758 | 0.452 | 0.817 | 0.425 | 0.222 | 0.830 | 0.738 | 0.862 |
|  | H_o_ | 0.159 | 0.129 | 0.169 | 0.188 | 0.381 | 0.088 | 0.017 | 0.385 | 0.422 | 0.477 |
|  | H_e_ | 0.151 | 0.176 | 0.501 | 0.224 | 0.457 | 0.208 | 0.110 | 0.408 | 0.491 | 0.546 |
|  | uH_e_ | 0.152 | 0.178 | 0.505 | 0.226 | 0.461 | 0.209 | 0.111 | 0.411 | 0.495 | 0.550 |
|  | F | -0.054 | 0.268 | 0.662 | 0.162 | 0.167 | 0.577 | 0.848 | 0.057 | 0.141 | 0.126 |
| San Salvador (Pillar Reef) | N | 68 | 69 | 69 | 68 | 46 | 66 | 67 | 61 | 66 | 69 |
|  | N_a_ | 43 | 10 | 9 | 21 | 5 | 3 | 3 | 12 | 2 | 3 |
|  | N_e_ | 18.459 | 1.297 | 4.174 | 6.917 | 2.771 | 2.176 | 1.094 | 1.395 | 1.062 | 1.076 |
|  | I | 3.330 | 0.624 | 1.709 | 2.408 | 1.288 | 0.845 | 0.203 | 0.765 | 0.136 | 0.180 |
|  | H_o_ | 0.838 | 0.159 | 0.609 | 0.794 | 0.283 | 0.606 | 0.060 | 0.230 | 0.061 | 0.072 |
|  | H_e_ | 0.946 | 0.229 | 0.760 | 0.855 | 0.639 | 0.541 | 0.086 | 0.283 | 0.059 | 0.070 |
|  | uH_e_ | 0.953 | 0.231 | 0.766 | 0.862 | 0.646 | 0.545 | 0.087 | 0.286 | 0.059 | 0.071 |
|  | F | 0.114 | 0.304 | 0.200 | 0.072 | 0.558 | -0.121 | 0.307 | 0.190 | -0.031 | -0.028 |
| Hog Cay, Exuma Sound | N | 35 | 37 | 36 | 37 | 18 | 37 | 22 | 34 | 35 | 37 |
|  | N_a_ | 9 | 2 | 4 | 6 | 3 | 1 | 2 | 5 | 4 | 3 |
|  | N_e_ | 3.186 | 1.027 | 1.882 | 3.303 | 2.667 | 1.000 | 1.541 | 1.163 | 2.579 | 1.468 |
|  | I | 1.505 | 0.072 | 0.865 | 1.380 | 1.028 | 0.000 | 0.536 | 0.361 | 1.046 | 0.583 |
|  | H_o_ | 0.600 | 0.027 | 0.167 | 0.595 | 0.167 | 0.000 | 0.091 | 0.147 | 0.571 | 0.297 |
|  | H_e_ | 0.686 | 0.027 | 0.469 | 0.697 | 0.625 | 0.000 | 0.351 | 0.140 | 0.612 | 0.319 |
|  | uH_e_ | 0.696 | 0.027 | 0.475 | 0.707 | 0.643 | 0.000 | 0.359 | 0.142 | 0.621 | 0.323 |
|  | F | 0.126 | -0.014 | 0.644 | 0.147 | 0.733 | #N/A | 0.741 | -0.049 | 0.067 | 0.068 |

N, sample size; Na,Number of alleles; Number of effective alleles; I, information index; Ho, observed heterozygosity; He, expected heterozygosity; uHe, unbiased expected heterozygosity; F, fixation index.

Table S2.Tests of Hardy-Weinberg Equilibrium within *A. elisabethae* collections for 7 loci at 11 sites in The Bahamas

| Site | Locus | df | Chi Sq. | p | Significance |
| --- | --- | --- | --- | --- | --- |
| Sweetings Cay | Pe_84 | 21 | 21.245 | 0.444 | ns |
|  | Pe_19 | 28 | 7.769 | 1.000 | ns |
|  | Pe_32 | 3 | 50.006 | 0.000 | *** |
|  | Pe_AG | 15 | 27.084 | 0.028 | * |
|  | AGAT3 | 36 | 38.241 | 0.368 | ns |
|  | Pe_AGC | 28 | 8.090 | 1.000 | ns |
|  | Pe_ATC | 10 | 5.067 | 0.887 | ns |
| Burrows South | Pe_84 | 21 | 11.468 | 0.953 | ns |
|  | Pe_19 | 10 | 41.186 | 0.000 | *** |
|  | Pe_32 | 6 | 1.246 | 0.975 | ns |
|  | Pe_AG | 10 | 11.530 | 0.318 | ns |
|  | AGAT3 | 21 | 30.993 | 0.074 | ns |
|  | Pe_AGC | 21 | 20.222 | 0.507 | ns |
|  | Pe_ATC | 10 | 10.186 | 0.424 | ns |
| Wood Cay | Pe_84 | 36 | 19.730 | 0.987 | ns |
|  | Pe_19 | 6 | 81.171 | 0.000 | *** |
|  | Pe_32 | Monomorphic | |  |  |
|  | Pe_AG | 6 | 9.160 | 0.165 | ns |
|  | AGAT3 | 45 | 66.360 | 0.021 | * |
|  | Pe_AGC | 21 | 20.229 | 0.507 | ns |
|  | Pe_ATC | 21 | 15.442 | 0.800 | ns |
| Mores Is. | Pe_84 | 10 | 23.860 | 0.008 | ** |
|  | Pe_19 | 15 | 4.590 | 0.995 | ns |
|  | Pe_32 | 1 | 0.005 | 0.946 | ns |
|  | Pe_AG | 6 | 0.681 | 0.995 | ns |
|  | AGAT3 | 21 | 29.375 | 0.105 | ns |
|  | Pe_AGC | 28 | 20.600 | 0.842 | ns |
|  | Pe_ATC | 21 | 9.308 | 0.987 | ns |
| Gorda Patch Reef | Pe_84 | 21 | 0.710 | 1.000 | ns |
|  | Pe_19 | 15 | 15.099 | 0.444 | ns |
|  | Pe_32 | Monomorphic | |  |  |
|  | Pe_AG | 3 | 29.700 | 0.000 | *** |
|  | AGAT3 | 21 | 68.109 | 0.000 | *** |
|  | Pe_AGC | 21 | 38.094 | 0.013 | * |
|  | Pe_ATC | 10 | 14.727 | 0.142 | ns |
| Cross Harbour Slope | Pe_84 | 3 | 3.554 | 0.314 | ns |
|  | Pe_19 | 3 | 2.875 | 0.411 | ns |
|  | Pe_32 | 1 | 33.000 | 0.000 | *** |
|  | Pe_AG | 3 | 16.486 | 0.001 | *** |
|  | AGAT3 | 15 | 2.547 | 1.000 | ns |
|  | Pe_AGC | 21 | 32.154 | 0.056 | ns |
|  | Pe_ATC | 6 | 15.555 | 0.016 | * |
| Cross Harbour Ridge | Pe_84 | 28 | 8.703 | 1.000 | ns |
|  | Pe_19 | 6 | 10.898 | 0.092 | ns |
|  | Pe_32 | Monomorphic | |  |  |
|  | Pe_AG | 6 | 6.304 | 0.390 | ns |
|  | AGAT3 | 15 | 7.381 | 0.946 | ns |
|  | Pe_AGC | 21 | 31.742 | 0.062 | ns |
|  | Pe_ATC | 10 | 10.637 | 0.386 | ns |
| Bimini | Pe_84 | 10 | 0.844 | 1.000 | ns |
|  | Pe_19 | 3 | 5.708 | 0.127 | ns |
|  | Pe_32 | 1 | 0.005 | 0.946 | ns |
|  | Pe_AG | 10 | 14.756 | 0.141 | ns |
|  | AGAT3 | 3 | 6.977 | 0.073 | ns |
|  | Pe_AGC | 28 | 69.484 | 0.000 | *** |
|  | Pe_ATC | 3 | 1.600 | 0.659 | ns |
| NW Tongue of the Ocean | Pe_84 | 28 | 27.685 | 0.481 | ns |
|  | Pe_19 | 10 | 10.479 | 0.399 | ns |
|  | Pe_32 | 6 | 54.132 | 0.000 | *** |
|  | Pe_AG | 10 | 15.596 | 0.112 | ns |
|  | AGAT3 | 21 | 23.535 | 0.316 | ns |
|  | Pe_AGC | 10 | 10.908 | 0.365 | ns |
|  | Pe_ATC | 6 | 5.561 | 0.474 | ns |
| Egg Is. | Pe_84 | 3 | 0.071 | 0.995 | ns |
|  | Pe_19 | 6 | 20.264 | 0.002 | ** |
|  | Pe_32 | 1 | 0.004 | 0.948 | ns |
|  | Pe_AG | 3 | 15.320 | 0.002 | ** |
|  | AGAT3 | 28 | 75.904 | 0.000 | *** |
|  | Pe_AGC | 21 | 27.229 | 0.163 | ns |
|  | Pe_ATC | 6 | 3.068 | 0.800 | ns |
| Andros | Pe_84 | 10 | 0.468 | 1.000 | ns |
|  | Pe_19 | 6 | 33.246 | 0.000 | *** |
|  | Pe_32 | 6 | 64.315 | 0.000 | *** |
|  | Pe_AG | 10 | 8.762 | 0.555 | ns |
|  | AGAT3 | 15 | 6.012 | 0.980 | ns |
|  | Pe_AGC | 3 | 4.517 | 0.211 | ns |
|  | Pe_ATC | 3 | 6.491 | 0.090 | ns |
| San Salvador | Pe_84 | 903 | 972.891 | 0.053 | ns |
|  | Pe_19 | 36 | 193.778 | 0.000 | *** |
|  | Pe_32 | 210 | 186.736 | 0.874 | ns |
|  | Pe_AG | 10 | 102.328 | 0.000 | *** |
|  | AGAT3 | 78 | 90.486 | 0.158 | ns |
|  | Pe_AGC | 1 | 0.064 | 0.800 | ns |
|  | Pe_ATC | 3 | 0.098 | 0.992 | ns |
| Hog Cay | Pe_84 | 36 | 18.577 | 0.993 | ns |
|  | Pe_19 | 6 | 60.038 | 0.000 | *** |
|  | Pe_32 | 15 | 17.601 | 0.284 | ns |
|  | Pe_AG | 3 | 18.960 | 0.000 | *** |
|  | AGAT3 | 10 | 0.214 | 1.000 | ns |
|  | Pe_AGC | 6 | 3.277 | 0.773 | ns |
|  | Pe_ATC | 3 | 0.980 | 0.806 | ns |
|  |  |  |  |  |  |
| **Key: ns=not significant, * P<0.05, ** P<0.01, *** P<0.001** | | | | |  |

Table S3. *Antillogorgia elisabethae* within population genetic equilibrium estimates corrected for multiple comparisons after 11700 permutations; adjusted p-value for 5% nominal level: 0.000085

|  | SC | BS | GB | MI | GP | CHSL | CHR | TR | TOTO | EGG | AN | PR | HC | All |
| --- | --- | --- | --- | --- | --- | --- | --- | --- | --- | --- | --- | --- | --- | --- |
| Pe_84 X Pe_62 | 1.0000 | 0.8688 | 0.9714 | 0.0995 | 0.8743 | 0.3865 | 0.2977 | 0.4656 | 1.0000 | 1.0000 | 0.1677 | 0.4392 | 0.2019 | 0.7034 |
| Pe_84 X Pe_19 | 0.6108 | 0.1216 | 0.1989 | 0.4886 | 0.3080 | 0.1338 | 0.3306 | 0.4336 | 0.1607 | 0.2161 | 0.1980 | 0.6323 | 0.3039 | 0.0285 |
| Pe_84 X Pe_32 | 0.6965 | 0.5920 | NA | 0.0886 | NA | 1.0000 | NA | 1.0000 | 0.3945 | 0.0692 | 0.8337 | 1.0000 | 0.6969 | 0.4342 |
| Pe_84 X Pe_AG | 0.3186 | 0.6389 | 0.8161 | 0.2820 | 1.0000 | 0.3159 | 0.5263 | 0.7593 | 0.4527 | 0.8433 | 0.6176 | 0.1998 | 0.9733 | 0.8469 |
| Pe_84 X AGAT1 | 0.3059 | 0.4895 | 0.2018 | 0.0222 | 0.8593 | 0.8069 | 0.8130 | 0.9355 | 0.0153 | 1.0000 | 0.5493 | 0.3611 | NA | 0.1563 |
| Pe_84 X AGAT2 | 0.0828 | 0.0256 | 0.6670 | 0.4562 | 1.0000 | 1.0000 | NA | NA | 0.2805 | NA | 1.0000 | 0.2680 | 0.6950 | 0.2550 |
| Pe_84 X AGAT3 | 0.6226 | 0.4026 | 0.8334 | 0.9310 | 0.7733 | 0.9026 | 0.0862 | 0.8615 | 0.8115 | 0.4596 | 0.0521 | 0.2371 | 0.9650 | 0.8089 |
| Pe_84 X Pe_AGC | 0.4781 | 0.1871 | 0.3581 | 0.8370 | 0.1568 | 0.3362 | 0.9286 | 0.5262 | 0.9994 | 0.9544 | 0.5555 | 0.3322 | 0.4685 | 0.8744 |
| Pe_84 X Pe_ATC | 0.1844 | 0.5727 | 0.2834 | 0.5741 | 0.2539 | 0.3507 | 0.3259 | 0.7416 | 0.4836 | 0.2696 | 0.8680 | 0.5290 | 0.3002 | 0.2361 |
| Pe_62 X Pe_19 | 0.2142 | 0.1127 | 0.6705 | 0.1044 | 0.5650 | 0.7554 | 0.5313 | 0.7535 | 0.0659 | 0.3210 | 0.6004 | 0.8095 | 1.0000 | 0.2349 |
| Pe_62 X Pe_32 | 0.4020 | 0.1459 | NA | 1.0000 | NA | 1.0000 | NA | 0.0886 | 0.7644 | 0.4455 | 0.3262 | 0.0530 | 0.3268 | 0.1029 |
| Pe_62 X Pe_AG | 0.9196 | 0.3115 | 0.5027 | 0.8080 | 0.2809 | 0.7804 | 0.1315 | 0.3188 | 0.9321 | 0.7805 | 0.3226 | 0.5337 | NA | 0.8143 |
| Pe_62 X AGAT1 | 0.6480 | 0.9913 | 0.0089 | 0.1052 | 0.1023 | 0.6786 | 0.0824 | 0.1265 | 0.6328 | 0.4119 | 0.7382 | 0.6268 | NA | 0.0737 |
| Pe_62 X AGAT2 | 0.7771 | 0.3587 | 0.9452 | 0.6189 | 0.2705 | 0.9044 | NA | NA | 0.5029 | NA | 0.2268 | 0.6017 | NA | 0.7915 |
| Pe_62 X AGAT3 | 0.0416 | 0.4704 | 0.7673 | 0.9556 | 0.6050 | 0.9823 | 0.2106 | 0.8822 | 0.9946 | 0.0348 | 0.7095 | 0.2043 | 1.0000 | 0.7340 |
| Pe_62 X Pe_AGC | 0.2312 | 0.7015 | 0.1533 | 0.0314 | 0.2619 | 0.8973 | 0.0408 | 0.7797 | 0.8918 | 0.6514 | 0.7188 | 0.3320 | 0.4874 | 0.2403 |
| Pe_62 X Pe_ATC | 0.6603 | 0.6787 | 0.8509 | 0.3164 | 0.0305 | 0.4968 | 0.2021 | 0.3650 | 0.0419 | 0.8575 | 0.9924 | 0.5886 | 1.0000 | 0.4465 |
| Pe_19 X Pe_32 | 0.1416 | 0.2975 | NA | 1.0000 | NA | 1.0000 | NA | 1.0000 | 0.4144 | 0.2971 | 0.0976 | 0.2185 | 0.4338 | 0.0871 |
| Pe_19 X Pe_AG | 0.9152 | 0.8925 | 0.6403 | 0.7668 | 0.7447 | 0.8330 | 0.1480 | 0.0872 | 0.5229 | 0.3639 | 0.0134 | 0.0706 | 0.5201 | 0.3972 |
| Pe_19 X AGAT1 | 0.2708 | 0.1426 | 0.2915 | 0.6540 | 0.2252 | 0.0292 | 0.1760 | 0.1475 | 0.3555 | 0.2238 | 0.1474 | 0.4010 | NA | 0.0169 |
| Pe_19 X AGAT2 | 0.5592 | 0.8934 | 0.5380 | 0.7955 | 0.1347 | 0.1206 | NA | NA | 0.6738 | NA | 0.6744 | 0.8721 | 0.4694 | 0.8362 |
| Pe_19 X AGAT3 | 0.0878 | 0.0732 | 0.2257 | 0.2131 | 0.1703 | 0.3977 | 0.0640 | 0.3743 | 0.3730 | 0.4167 | 0.8639 | 0.1550 | 0.8650 | 0.0202 |
| Pe_19 X Pe_AGC | 0.0763 | 0.5895 | 0.1807 | 0.8506 | 0.1692 | 0.4499 | 0.1306 | 0.5052 | 0.6913 | 0.0903 | 0.2756 | 0.9778 | 0.0174 | 0.0783 |
| Pe_19 X Pe_ATC | 0.5530 | 0.2540 | 0.6936 | 0.6826 | 0.8003 | 0.7444 | 0.9256 | 0.1442 | 0.5349 | 0.0680 | 0.1041 | 0.0004 | 0.2273 | 0.1844 |
| Pe_32 X Pe_AG | 0.8819 | 0.2654 | NA | 0.1459 | NA | 0.0423 | NA | 0.1997 | 0.0490 | 1.0000 | 0.2391 | 0.1027 | 0.3996 | 0.0369 |
| Pe_32 X AGAT1 | 0.8134 | 0.8221 | NA | NA | NA | 0.7498 | NA | 0.1799 | 0.4175 | 1.0000 | 0.2155 | 0.5907 | NA | 0.6785 |
| Pe_32 X AGAT2 | 0.7963 | 0.1059 | NA | NA | NA | 1.0000 | NA | NA | 0.3148 | NA | 0.2586 | 0.6448 | 0.6032 | 0.3595 |
| Pe_32 X AGAT3 | 0.3060 | 0.4544 | NA | 1.0000 | NA | 0.1800 | NA | 0.6036 | 0.4900 | 0.5377 | 0.8393 | 0.7042 | 0.3639 | 0.5580 |
| Pe_32 X Pe_AGC | 0.0056 | 0.8918 | NA | 1.0000 | NA | 0.8741 | NA | 0.3073 | 0.1839 | 0.9025 | 0.1568 | 0.5075 | 0.7801 | 0.3046 |
| Pe_32 X Pe_ATC | 0.2209 | 0.5307 | NA | 0.6327 | NA | 0.3471 | NA | 1.0000 | 0.9260 | 0.2699 | 0.7486 | 0.4987 | 0.4276 | 0.5393 |
| Pe_AG X AGAT1 | 0.1939 | 0.6257 | 0.0301 | 0.2810 | 0.1262 | 0.6268 | 0.8639 | 0.4042 | 0.2656 | 0.4597 | 0.4162 | 0.1736 | NA | 0.0667 |
| Pe_AG X AGAT2 | 0.4480 | 0.7074 | 0.4556 | 0.1097 | 0.8692 | 0.6968 | NA | NA | 0.8682 | NA | 0.5777 | 0.1203 | 0.8331 | 0.6346 |
| Pe_AG X AGAT3 | 0.3983 | 0.8774 | 0.0614 | 0.2226 | 0.9727 | 0.2005 | 0.0162 | 0.6437 | 0.0233 | 0.1513 | 0.7145 | 0.9775 | 0.9727 | 0.3722 |
| Pe_AG X Pe_AGC | 0.3151 | 0.5691 | 0.7707 | 0.4341 | 0.6180 | 0.4266 | 0.7221 | 0.4733 | 0.5015 | 0.1104 | 0.4265 | 0.0609 | 1.0000 | 0.4590 |
| Pe_AG X Pe_ATC | 0.0345 | 0.0903 | 0.9204 | 0.9248 | 0.6523 | 0.0682 | 0.3197 | 0.0913 | 0.5683 | 0.1829 | 0.3456 | 0.6961 | 0.5392 | 0.1600 |
| AGAT1 X AGAT2 | 0.4468 | 0.6147 | 0.8322 | 0.2139 | 0.7580 | 0.4633 | NA | NA | 0.9681 | NA | 0.5431 | 0.1802 | NA | 0.7469 |
| AGAT1 X AGAT3 | 0.2402 | 0.3778 | 0.1165 | 0.8486 | 0.4882 | 0.7569 | 0.7863 | 0.8543 | 0.6939 | 0.9984 | 0.9855 | 0.2330 | NA | 0.9480 |
| AGAT1 X Pe_AGC | 0.9362 | 0.1604 | 0.2242 | 0.9163 | 0.4727 | 0.1040 | 0.9585 | 0.1857 | 0.4225 | 0.4977 | 0.3317 | 0.6441 | NA | 0.6040 |
| AGAT1 X Pe_ATC | 0.2610 | 0.8183 | 0.6359 | 0.0094 | 0.2714 | 0.6827 | 0.4805 | 0.0373 | 0.3933 | 0.6025 | 0.6865 | 0.5223 | NA | 0.1942 |
| AGAT2 X AGAT3 | 0.4245 | 0.8258 | 0.0329 | 0.4131 | 0.6770 | 0.9379 | NA | NA | 0.8157 | NA | 0.9452 | 0.7252 | 0.8804 | 0.9047 |
| AGAT2 X Pe_AGC | 0.8152 | 0.5039 | 0.8360 | 0.4416 | 0.3865 | 0.8220 | NA | NA | 0.1057 | NA | 0.9584 | 0.5015 | 0.4226 | 0.7236 |
| AGAT2 X Pe_ATC | 0.5181 | 0.0593 | 0.9651 | 0.4379 | 0.8879 | 0.7432 | NA | NA | 0.8061 | NA | 0.3109 | 0.1568 | 0.3387 | 0.6032 |
| AGAT3 X Pe_AGC | 0.2180 | 0.2743 | 0.9039 | 0.0769 | 0.6658 | 0.9521 | 0.3071 | 0.1492 | 0.4827 | 0.5950 | 0.9613 | 0.3325 | 0.6021 | 0.4165 |
| AGAT3 X Pe_ATC | 0.8861 | 0.2886 | 0.6640 | 0.4948 | 0.8830 | 0.1982 | 0.6639 | 0.5879 | 0.5972 | 0.2164 | 0.2630 | 0.5685 | 0.8166 | 0.7210 |
| Pe_AGC X Pe_ATC | 0.3350 | 0.7601 | 0.4007 | 0.1527 | 0.9642 | 0.6143 | 0.6974 | 0.0758 | 0.3627 | 0.3600 | 0.8031 | 1.0000 | 0.2934 | 0.3750 |

Table S4. *Antillogorgia elisabethae* pairwise F*_ST_* values calculated in FreeNA with and without the ENA correction described in Chapuis and Estoup (2007)

| F*_ST_* with ENA corrections | | |  |  |  |  |  |  |  |  |  |  |
| --- | --- | --- | --- | --- | --- | --- | --- | --- | --- | --- | --- | --- |
| Population | SC | BS | GB | MI | GP | CHSL | CHR | TR | TOTO | EGG | AN | PR |
| BS | 0.0118 |  |  |  |  |  |  |  |  |  |  |  |
| GB | 0.0761 | 0.0642 |  |  |  |  |  |  |  |  |  |  |
| MI | 0.0315 | 0.0266 | 0.0638 |  |  |  |  |  |  |  |  |  |
| GP | 0.0393 | 0.0209 | 0.0492 | 0.0068 |  |  |  |  |  |  |  |  |
| CHSL | 0.0418 | 0.0269 | 0.0679 | 0.0200 | 0.0095 |  |  |  |  |  |  |  |
| CHR | 0.0626 | 0.0488 | 0.0895 | 0.0370 | 0.0243 | -0.0004 |  |  |  |  |  |  |
| TR | 0.1169 | 0.0878 | 0.1440 | 0.1351 | 0.0971 | 0.0866 | 0.0850 |  |  |  |  |  |
| TOTO | 0.1402 | 0.1334 | 0.1314 | 0.1169 | 0.0913 | 0.0983 | 0.1192 | 0.1795 |  |  |  |  |
| EGG | 0.1068 | 0.0975 | 0.1267 | 0.0461 | 0.0399 | 0.0370 | 0.0391 | 0.1625 | 0.1025 |  |  |  |
| AN | 0.0802 | 0.0897 | 0.0879 | 0.0669 | 0.0605 | 0.0490 | 0.0637 | 0.1544 | 0.0646 | 0.0698 |  |  |
| PR | 0.3850 | 0.3717 | 0.3236 | 0.4247 | 0.3833 | 0.4131 | 0.4542 | 0.4319 | 0.3745 | 0.4981 | 0.4213 |  |
| HC | 0.2466 | 0.2682 | 0.2257 | 0.2723 | 0.2743 | 0.2927 | 0.3325 | 0.4046 | 0.2810 | 0.3513 | 0.2813 | 0.2741 |
|  |  |  |  |  |  |  |  |  |  |  |  |  |
| F*_ST_* without ENA corrections | | |  |  |  |  |  |  |  |  |  |  |
| Population | SC | BS | GB | MI | GP | CHSL | CHR | TR | TOTO | EGG | AN | PR |
| BS | 0.0170 |  |  |  |  |  |  |  |  |  |  |  |
| GB | 0.0697 | 0.0575 |  |  |  |  |  |  |  |  |  |  |
| MI | 0.0293 | 0.0260 | 0.0630 |  |  |  |  |  |  |  |  |  |
| GP | 0.0449 | 0.0245 | 0.0493 | 0.0061 |  |  |  |  |  |  |  |  |
| CHSL | 0.0379 | 0.0158 | 0.0632 | 0.0111 | 0.0016 |  |  |  |  |  |  |  |
| CHR | 0.0543 | 0.0347 | 0.0880 | 0.0299 | 0.0218 | -0.0030 |  |  |  |  |  |  |
| TR | 0.1248 | 0.0877 | 0.1524 | 0.1461 | 0.1065 | 0.0921 | 0.0965 |  |  |  |  |  |
| TOTO | 0.1520 | 0.1524 | 0.1487 | 0.1292 | 0.1008 | 0.1019 | 0.1241 | 0.1957 |  |  |  |  |
| EGG | 0.1023 | 0.0961 | 0.1340 | 0.0376 | 0.0376 | 0.0363 | 0.0425 | 0.1863 | 0.1092 |  |  |  |
| AN | 0.0882 | 0.1016 | 0.0990 | 0.0670 | 0.0681 | 0.0484 | 0.0610 | 0.1695 | 0.0749 | 0.0717 |  |  |
| PR | 0.4055 | 0.3894 | 0.3437 | 0.4447 | 0.3975 | 0.4321 | 0.4651 | 0.4380 | 0.4025 | 0.5184 | 0.4569 |  |
| HC | 0.2545 | 0.2682 | 0.2120 | 0.2774 | 0.2737 | 0.3059 | 0.3295 | 0.4099 | 0.3108 | 0.3614 | 0.3065 | 0.2849 |

Table S5.*G”*_ST_ values for pairwise comparisons of adult populations of *A. elisabethae* in The Bahamas. *G*’’_ST_ values are presented below the diagonal and probability of the value equaling 0.0 is presented above the diagonal. Significant (*p* < 0.05) *G*’’_ST_ values are also denoted in bold.

|  | **WC** | **SC** | **BS** | **MI** | **GP** | **CHSL** | **CHR** | **EGG** | **TOTO** | **AN** | **TR** | **HC** | **PR** |
| --- | --- | --- | --- | --- | --- | --- | --- | --- | --- | --- | --- | --- | --- |
| **WC** |  | 0.001 | 0.001 | 0.001 | 0.001 | 0.001 | 0.001 | 0.001 | 0.001 | 0.001 | 0.001 | 0.001 | 0.001 |
| **SC** | **0.141** |  | 0.051 | 0.001 | 0.001 | 0.002 | 0.001 | 0.001 | 0.001 | 0.001 | 0.001 | 0.001 | 0.001 |
| **BS** | **0.147** | 0.015 |  | 0.003 | 0.002 | 0.070 | 0.001 | 0.001 | 0.001 | 0.001 | 0.001 | 0.001 | 0.001 |
| **MI** | **0.117** | **0.056** | **0.029** |  | 0.314 | 0.027 | 0.001 | 0.001 | 0.001 | 0.001 | 0.001 | 0.001 | 0.001 |
| **GP** | **0.120** | **0.085** | **0.059** | 0.003 |  | 0.195 | 0.029 | 0.310 | 0.001 | 0.001 | 0.001 | 0.001 | 0.001 |
| **CHSL** | **0.143** | **0.036** | 0.016 | **0.021** | 0.010 |  | 0.459 | 0.001 | 0.001 | 0.001 | 0.001 | 0.001 | 0.001 |
| **CHR** | **0.159** | **0.049** | **0.037** | **0.035** | **0.019** | -0.001 |  | 0.001 | 0.001 | 0.001 | 0.001 | 0.001 | 0.001 |
| **EGG** | **0.187** | **0.145** | **0.101** | **0.047** | 0.004 | **0.051** | **0.052** |  | 0.001 | 0.001 | 0.001 | 0.001 | 0.001 |
| **TOTO** | **0.300** | **0.305** | **0.285** | **0.246** | **0.178** | **0.191** | **0.213** | **0.173** |  | 0.001 | 0.001 | 0.001 | 0.001 |
| **AN** | **0.153** | **0.146** | **0.161** | **0.126** | **0.101** | **0.095** | **0.106** | **0.134** | **0.129** |  | 0.001 | 0.001 | 0.001 |
| **TR** | **0.270** | **0.100** | **0.121** | **0.152** | **0.136** | **0.085** | **0.067** | **0.144** | **0.256** | **0.160** |  | 0.001 | 0.001 |
| **HC** | **0.411** | **0.505** | **0.484** | **0.478** | **0.493** | **0.500** | **0.532** | **0.546** | **0.539** | **0.496** | **0.620** |  | 0.001 |
| **PR** | **0.672** | **0.747** | **0.742** | **0.758** | **0.748** | **0.727** | **0.771** | **0.786** | **0.648** | **0.695** | **0.818** | **0.407** |  |
| **G''st values below the diagonal. Probability, P(rand >= data) based on 999 permutations is shown above diagonal.** | | | | | | | | | | |  |  |  |

Table S6. *G”*_ST_ values for pairwise comparisons of adult and recruit populations of *A. elisabethae* in The Bahamas. *G*’’_ST_ values are presented below the diagonal and probability of the value equaling 0.0 is presented above the diagonal. Significant (*p* < 0.05) *G*’’_ST_ values are also denoted in bold. Shaded blocks depict adults and recruits from the same site.

|  | **BS-A** | **BS-R-2004** | **BS-R-2009** | **BS-R-2012** | **AB-A** | **AB-R-2004** | **AB-R-2005** | **AB-R-2009** | **AB-R-2012** | **CH60-A** | **CH60-R-2009** | **CH60-R-2012** | **CHR-A** | **CHR-R-2004** | **CHR-R-2007** | **CHR-R-2009** | **CHR-R-2010** | **CHR-R-2011** | **CHR-R-2012** |
| --- | --- | --- | --- | --- | --- | --- | --- | --- | --- | --- | --- | --- | --- | --- | --- | --- | --- | --- | --- |
| **BS-A** |  | 0.057 | 0.753 | 0.952 | 0.001 | 0.041 | 0.003 | 0.076 | 0.158 | 0.138 | 0.002 | 0.001 | 0.010 | 0.375 | 0.034 | 0.002 | 0.001 | 0.001 | 0.001 |
| **BS-R-2004** | 0.026 |  | 0.026 | 0.090 | 0.001 | 0.004 | 0.005 | 0.047 | 0.047 | 0.027 | 0.003 | 0.001 | 0.002 | 0.057 | 0.045 | 0.004 | 0.002 | 0.004 | 0.005 |
| **BS-R-2009** | -0.010 | **0.054** |  | 0.823 | 0.006 | 0.025 | 0.004 | 0.052 | 0.196 | 0.133 | 0.002 | 0.001 | 0.004 | 0.122 | 0.022 | 0.003 | 0.003 | 0.001 | 0.002 |
| **BS-R-2012** | -0.021 | 0.031 | -0.018 |  | 0.019 | 0.128 | 0.007 | 0.247 | 0.331 | 0.454 | 0.008 | 0.002 | 0.082 | 0.620 | 0.151 | 0.030 | 0.049 | 0.016 | 0.029 |
| **AB-A** | 0.056 | 0.108 | 0.060 | 0.047 |  | 0.939 | 0.175 | 0.749 | 0.893 | 0.188 | 0.058 | 0.033 | 0.047 | 0.014 | 0.105 | 0.056 | 0.144 | 0.172 | 0.026 |
| **AB-R-2004** | 0.041 | 0.099 | 0.059 | 0.031 | -0.026 |  | 0.317 | 0.889 | 0.672 | 0.497 | 0.225 | 0.380 | 0.449 | 0.142 | 0.380 | 0.330 | 0.340 | 0.487 | 0.506 |
| **AB-R-2005** | 0.087 | 0.120 | 0.107 | 0.088 | 0.019 | 0.012 |  | 0.292 | 0.247 | 0.044 | 0.169 | 0.090 | 0.095 | 0.021 | 0.188 | 0.121 | 0.082 | 0.252 | 0.148 |
| **AB-R-2009** | 0.027 | 0.042 | 0.037 | 0.012 | -0.013 | -0.029 | 0.011 |  | 0.744 | 0.685 | 0.420 | 0.271 | 0.496 | 0.209 | 0.675 | 0.638 | 0.614 | 0.691 | 0.892 |
| **AB-R-2012** | 0.028 | 0.058 | 0.022 | 0.010 | -0.031 | -0.016 | 0.022 | -0.025 |  | 0.473 | 0.282 | 0.189 | 0.211 | 0.196 | 0.477 | 0.332 | 0.482 | 0.600 | 0.413 |
| **CH60-A** | 0.011 | 0.049 | 0.017 | -0.001 | 0.011 | -0.005 | 0.044 | -0.011 | -0.003 |  | 0.047 | 0.036 | 0.496 | 0.444 | 0.719 | 0.326 | 0.463 | 0.306 | 0.364 |
| **CH60-R-2009** | 0.064 | 0.071 | 0.086 | 0.050 | 0.024 | 0.015 | 0.019 | 0.000 | 0.014 | **0.020** |  | 0.809 | 0.440 | 0.083 | 0.790 | 0.701 | 0.617 | 0.720 | 0.298 |
| **CH60-R-2012** | 0.074 | 0.091 | 0.104 | 0.060 | 0.025 | 0.002 | 0.026 | 0.005 | 0.023 | **0.020** | -0.006 |  | 0.525 | 0.036 | 0.721 | 0.352 | 0.087 | 0.285 | 0.234 |
| **CHR-A** | 0.031 | 0.061 | 0.057 | 0.020 | 0.022 | -0.001 | 0.024 | -0.002 | 0.018 | -0.002 | -0.001 | -0.001 |  | 0.161 | 0.984 | 0.914 | 0.792 | 0.734 | 0.742 |
| **CHR-R-2004** | 0.002 | 0.038 | 0.025 | -0.006 | 0.058 | 0.035 | 0.073 | 0.017 | 0.033 | -0.001 | 0.025 | 0.030 | 0.013 |  | 0.566 | 0.125 | 0.098 | 0.085 | 0.091 |
| **CHR-R-2007** | 0.027 | 0.036 | 0.045 | 0.017 | 0.018 | 0.003 | 0.019 | -0.011 | -0.002 | -0.009 | -0.010 | -0.006 | -0.015 | -0.005 |  | 0.998 | 0.952 | 0.929 | 0.917 |
| **CHR-R-2009** | 0.040 | 0.049 | 0.060 | 0.031 | 0.018 | 0.005 | 0.019 | -0.007 | 0.006 | 0.002 | -0.004 | 0.001 | -0.007 | 0.015 | -0.016 |  | 0.774 | 0.873 | 0.839 |
| **CHR-R-2010** | 0.036 | 0.060 | 0.049 | 0.024 | 0.010 | 0.006 | 0.027 | -0.006 | -0.004 | 0.000 | -0.003 | 0.007 | -0.005 | 0.016 | -0.011 | -0.003 |  | 0.826 | 0.302 |
| **CHR-R-2011** | 0.042 | 0.062 | 0.065 | 0.034 | 0.009 | -0.003 | 0.011 | -0.007 | -0.009 | 0.003 | -0.005 | 0.002 | -0.005 | 0.019 | -0.011 | -0.004 | -0.004 |  | 0.619 |
| **CHR-R-2012** | 0.039 | 0.052 | 0.055 | 0.027 | 0.022 | -0.002 | 0.017 | -0.015 | 0.001 | 0.001 | 0.003 | 0.003 | -0.004 | 0.018 | -0.010 | -0.004 | 0.001 | -0.002 |  |
| **G''st values below the diagonal. Probability, P(rand >= data) based on 999 permutations is shown above diagonal.** | | | | | | | | | | | | | |  |  |  |  |  |  |

Table S7 -AMOVA of *A.elisabethae* Adults and Recruits from Cross Harbour

**Adults vs Recruits^1^ Sum of Variance Percentage**

**d.f. squares components of variation P**

Among groups 1 0.772 -0.001 -0.05 0.785

Among sites

within groups 8 7.191 -0.002 -0.14 0.967

Within

populations 1474 1668.511 1.132 100.19 0.972

Total 1483 1676.474 1.130

^1^Adults(CHR-adults, CHSL-adults) vs. Recruits (CHR- recruits -2007, CHR- recruits -2004, CHSL- recruits -2009, CHR- recruits -2009, CHR- recruits -2011, CHR- recruits -2010, CHR- recruits -2012, CHSL- recruits -2012)

**CHR vs CHSL ^2^ Sum of Variance Percentage P**

**d.f. squares components of variation**

Among sites 1 1.914 0.002 0.19 0.081

Among years

within sites 8 6.048 -0.0023 -0.23 0.997

Within populations 1474 1668.511 1.132 100.04 0.980

Total 1483 1676.474 1.131

^2^CHR(CHR-adults, CHR-recruits-2007, CHR- recruits -2004, CHR- recruits -2009, CHR- recruits -2011, CHR- recruits -2010, CHR- recruits -2012,) vs. CHSL (CHSL-adults, CHSL- recruits -2009, CHSL- recruits -2012

Table S8.Geneclass2 assignment of *A. elisabethae* recruits.

|  |  | GeneClass2 Best Assignment (proportion of recruits)* | | | | | | | | | | | | | N |
| --- | --- | --- | --- | --- | --- | --- | --- | --- | --- | --- | --- | --- | --- | --- | --- |
| Source | Year | GB | SC | BS | MI | GP | CHSL | CHR | EGG | TOTO | AN1 | TR | HC | PR |  |
| GP | 2004 | 0.20 | 0.13 |  |  | **0.67** |  |  |  |  |  |  |  |  | 15 |
|  | 2005 | 0.13 | 0.07 |  | 0.07 | **0.53** |  | 0.20 |  |  |  |  |  |  | 15 |
|  | 2009 | 0.05 | 0.10 |  |  | **0.29** | 0.10 | 0.24 |  | 0.10 |  | 0.14 |  |  | 21 |
|  | 2012 | 0.10 | 0.10 |  |  | **0.70** | 0.10 |  |  |  |  |  |  |  | 10 |
|  | Total | 0.11 | 0.10 |  | 0.02 | **0.51** | 0.05 | 0.13 |  | 0.03 |  | 0.05 |  |  | 61 |
| BS | 2004 | 0.05 | 0.38 | **0.19** | 0.10 | 0.14 |  | 0.10 | 0.05 |  |  |  |  |  | 21 |
|  | 2009 | 0.04 | 0.44 | **0.24** | 0.04 | 0.12 | 0.04 | 0.04 |  | 0.04 |  |  |  |  | 25 |
|  | 2012 | 0.04 | 0.29 | **0.29** | 0.17 | 0.13 | 0.04 |  |  | 0.04 |  |  |  |  | 24 |
|  | Total | 0.04 | 0.37 | **0.24** | 0.10 | 0.13 | 0.03 | 0.04 | 0.01 | 0.03 |  |  |  |  | 70 |
| CHSL | 2009 | 0.11 | 0.11 | 0.09 |  | 0.36 | **0.04** | 0.11 |  | 0.04 |  | 0.13 |  |  | 45 |
|  | 2012 | 0.10 | 0.11 | 0.12 | 0.04 | 0.26 | **0.07** | 0.22 | 0.02 | 0.03 |  | 0.03 |  |  | 92 |
|  | Total | 0.10 | 0.11 | 0.11 | 0.03 | 0.29 | **0.06** | 0.18 | 0.01 | 0.04 |  | 0.07 |  |  | 137 |
| CHR | 2004 | 0.13 | 0.08 | 0.21 | 0.17 | 0.13 | 0.21 |  |  |  | 0.04 | 0.04 |  |  | 24 |
|  | 2007 | 0.03 | 0.19 | 0.11 | 0.05 | 0.30 | 0.05 | **0.19** | 0.03 |  |  | 0.05 |  |  | 37 |
|  | 2009 | 0.07 | 0.20 | 0.07 | 0.04 | 0.26 | 0.06 | **0.22** | 0.01 | 0.04 | 0.01 | 0.01 |  |  | 134 |
|  | 2010 | 0.05 | 0.22 | 0.07 | 0.06 | 0.30 | 0.07 | **0.11** | 0.04 | 0.04 |  | 0.04 |  |  | 108 |
|  | 2011 | 0.09 | 0.09 | 0.12 | 0.04 | 0.27 | 0.03 | **0.20** | 0.02 | 0.08 | 0.01 | 0.05 |  |  | 100 |
|  | 2012 | 0.07 | 0.12 | 0.11 | 0.06 | 0.36 | 0.08 | **0.10** | 0.01 | 0.03 | 0.02 | 0.04 |  |  | 105 |
|  | Total | 0.07 | 0.16 | 0.10 | 0.06 | 0.29 | 0.07 | **0.16** | 0.02 | 0.04 | 0.01 | 0.04 |  |  | 508 |

*Bold denotes recruits assigned to the source site. Site abbreviations as in Table 1.

Table S9. Proportions of colonies (adults and recruits) at a site (destination) originating from the potential source sites. Values are means (std. dev.) based on 5 BayesAss analyses.

| **SOURCE** | **WC** | **SC** | **BS** | **MI** | **GP** | **CHSL** | **CHR** | **EGG** | **TOTO** | **AN** | **TR** | **HC** | **PR** | **Self**  **Recruits** | **Migrants** |
| --- | --- | --- | --- | --- | --- | --- | --- | --- | --- | --- | --- | --- | --- | --- | --- |
| **DESTINATION** |  |  |  |  |  |  |  |  |  |  |  |  |  |  |  |
| **Wood Cay (WC)** | **0.878*** | 0.012 | 0.012 | 0.011 | 0.005 | 0.005 | 0.026 | 0.007 | 0.006 | 0.017 | 0.011 | 0.007 | 0.005 | 0.878 | 0.122 |
|  | **(0.025)** | (0.018) | (0.020) | 0.018) | (0.005) | (0.005) | 0.024) | (0.007) | (0.006) | (0.015) | (0.010) | (0.006) | (0.005) |  |  |
| **Sweeting Cay** | 0.008 | **0.723*** | 0.053 | 0.052 | 0.005 | 0.005 | 0.102 | 0.007 | 0.006 | 0.015 | 0.014 | 0.007 | 0.005 | 0.723 | 0.277 |
| **(SC)** | (0.006) | (0.087) | (0.096) | (0.093) | (0.005) | (0.005) | 0.103) | (0.008) | (0.006) | (0.013) | (0.013) | (0.007) | (0.005) |  |  |
| **Burrows South**  **(BS)** | 0.011 | 0.040 | **0.700*** | 0.035 | 0.005 | 0.005 | 0.150 | 0.009 | 0.006 | 0.011 | 0.018 | 0.006 | 0.005 | 0.700 | 0.300 |
|  | (0.007) | (0.059) | (0.059) | (0.061) | (0.005) | (0.005) | 0.080) | (0.010) | (0.006) | (0.011) | (0.014) | (0.006) | (0.005) |  |  |
| **Mores Is (MI)** | 0.007 | 0.028 | 0.033 | **0.689*** | 0.005 | 0.005 | **0.175*** | 0.022 | 0.007 | 0.010 | 0.009 | 0.006 | 0.005 | 0.689 | 0.311 |
|  | (0.005) | (0.046) | (0.058) | (0.039) | (0.005) | (0.005) | 0.070) | (0.029) | (0.008) | (0.010) | (0.009) | (0.005) | (0.005) |  |  |
| **Gorda Patch (GP)** | 0.012 | 0.011 | 0.012 | 0.010 | **0.675*** | 0.008 | **0.175*** | 0.037 | 0.012 | 0.018 | 0.014 | 0.009 | 0.008 | 0.675 | 0.325 |
|  | (0.008) | (0.012) | (0.015) | (0.011) | (0.008) | (0.008) | 0.053) | (0.048) | (0.012) | (0.015) | (0.013) | (0.009) | (0.008) |  |  |
| **Cross Harbour Slope (CHSL)** | 0.008 | 0.011 | 0.012 | 0.009 | 0.006 | **0.673*** | **0.195*** | 0.021 | 0.015 | 0.017 | 0.019 | 0.007 | 0.006 | 0.673 | 0.327 |
|  | (0.006) | (0.013) | (0.023) | (0.012) | (0.006) | (0.006) | 0.046) | (0.031) | (0.014) | (0.013) | (0.015) | (0.007) | (0.006) |  |  |
| **Cross Harbour Ridge (CHR)** | 0.007 | 0.011 | 0.013 | 0.010 | 0.005 | 0.005 | **0.825*** | 0.029 | 0.018 | 0.021 | 0.046 | 0.006 | 0.005 | 0.825 | 0.175 |
|  | (0.005) | (0.015) | (0.025) | (0.015) | (0.005) | (0.005) | 0.058) | (0.050) | (0.017) | (0.019) | (0.026) | (0.006) | (0.005) |  |  |
| **Egg Is (EGG)** | 0.005 | 0.005 | 0.006 | 0.007 | 0.004 | 0.004 | **0.187*** | **0.724*** | 0.010 | 0.018 | 0.020 | 0.005 | 0.004 | 0.724 | 0.276 |
|  | (0.004) | (0.005) | (0.009) | (0.010) | (0.004) | (0.004) | 0.093) | (0.083) | (0.010) | (0.017) | (0.021) | (0.005) | (0.004) |  |  |
| **Tongue of the Ocean (ToTO)** | 0.007 | 0.007 | 0.006 | 0.006 | 0.005 | 0.005 | 0.014 | 0.008 | **0.769*** | 0.145 | 0.008 | 0.015 | 0.005 | 0.769 | 0.231 |
|  | (0.005) | (0.007) | (0.006) | (0.007) | (0.005) | (0.005) | 0.014) | (0.010) | (0.083) | (0.093) | (0.008) | (0.012) | (0.005) |  |  |
| **Andros Is (AN)** | 0.006 | 0.006 | 0.006 | 0.005 | 0.004 | 0.004 | 0.011 | 0.005 | 0.013 | **0.921*** | 0.009 | 0.005 | 0.004 | 0.921 | 0.079 |
|  | (0.005) | (0.007) | (0.008) | (0.006) | (0.004) | (0.004) | 0.010) | (0.006) | (0.014) | (0.023) | (0.008) | (0.005) | (0.004) |  |  |
| **Triangle Rock (TR)** | 0.007 | 0.006 | 0.006 | 0.007 | 0.005 | 0.005 | 0.039 | 0.018 | 0.009 | 0.018 | **0.871*** | 0.005 | 0.005 | 0.871 | 0.129 |
|  | (0.005) | (0.007) | (0.007) | (0.009) | (0.005) | (0.005) | 0.035) | (0.027) | (0.008) | (0.014) | (0.041) | (0.005) | (0.005) |  |  |
| **Hog Cay (HC)** | 0.009 | 0.007 | 0.006 | 0.006 | 0.006 | 0.006 | 0.010 | 0.007 | 0.009 | 0.012 | 0.007 | **0.908*** | 0.006 | 0.908 | 0.092 |
|  | (0.006) | (0.007) | (0.006) | (0.006) | (0.006) | (0.006) | 0.009) | (0.007) | (0.009) | (0.011) | (0.007) | (0.022) | (0.006) |  |  |
| **Pillar Reef (PR)** | 0.004 | 0.004 | 0.004 | 0.004 | 0.004 | 0.004 | 0.004 | 0.004 | 0.005 | 0.004 | 0.004 | 0.005 | **0.949*** | 0.949 | 0.051 |
|  | (0.003) | (0.004) | (0.004) | (0.004) | (0.004) | (0.004) | 0.004) | 0.004 | (0.005) | (0.004) | (0.004) | (0.005) | (0.013) |  |  |

- * Value significantly greater than 0.0 (P<0.05).

Figure S1 -Relationship between genetic distance and geographic distance among samples from the Little Bahama Bank. *F*_ST_ based on 6 loci. Regression line is for the combined data set.

Fig S2 - Upper panels, Evanno’s DeltaK and Ln(K) for Structure analysis of *A. elisabethae* adult colonies from 13 sites using 7 microsatellite analyses. Lower panels, Evanno’s DeltaK and Ln(K) for Structure analysis of *A. elisabethae* adult colonies from 11 sites using 7 microsatellite analyses.
